# Genetic Relatedness and Heterotic Grouping in MRIZP Elite Maize Inbred Lines Using SNP Markers from 25k SNP Array and RNA-seq Data

**DOI:** 10.3390/cimb48060586

**Published:** 2026-06-02

**Authors:** Marko Mladenović, Bojana Banović Đeri, Ana Nikolić, Dragana Dudić, Slaven Prodanović, Sanja Z. Perić, Nikola Grčić

**Affiliations:** 1 Maize Research Institute “Zemun Polje”, 11185 Belgrade, Serbia; mmladenovic@mrizp.rs (M.M.); anikolic@mrizp.rs (A.N.); speric@mrizp.rs (S.Z.P.); 2 Institute of Molecular Genetics and Genetic Engineering, University of Belgrade, 11042 Belgrade, Serbia; bojana.banovic@imgge.bg.ac.rs; 3 Faculty of Mathematics and Computer Sciences, Alfa BK University, 11070 Belgrade, Serbia; dragana.dudic@alfa.edu.rs; 4 Faculty of Agriculture, University of Belgrade, 11080 Belgrade, Serbia; slavenp@agrif.bg.ac.rs

**Keywords:** maize inbred lines, heterotic groups, population structure, genetic relatedness, 25k SNP array, RNA-seq

## Abstract

Knowledge of population structure and genetic relationship among inbred lines is essential for exploiting heterosis in maize breeding programs. This study evaluated the concordance between 25k Illumina^®^ Infinum Maize SNP (Single Nucleotide Polymorphism) Array (25k SNP array)-derived and ribonucleic acid (RNA)-sequencing (seq)-derived markers in estimating genetic relatedness and population structure within the Maize Research Institute “Zemun Polje” (MRIZP) breeding program. A panel of 28 elite MRIZP maize inbred lines, along with two public lines, was analyzed. For the RNA-seq data, three alternative SNP datasets were generated based on heterozygous-site handling (ALL, HOM, and FINAL) to assess their impact on downstream genetic inference. These approaches had distinct effects on clustering resolution and genetic relationship structure. The FINAL dataset, in which heterozygous positions were recoded as missing values and re-filtered, was selected as the most balanced dataset for comparative analyses with 25k SNP array data. Only a limited number of overlapping SNP positions were identified between RNA-seq and 25k SNP array datasets (six in ALL, two in FINAL, and none in HOM), all located within coding regions. Despite this minimal overlap, distance-based analyses revealed partial concordance in genetic relationship patterns and population structure between platforms. Genetic distances estimated from 25k SNP array markers were consistent with pedigree records and provided more informative insights than pedigree data alone. Population structure inferred from 25k SNP array data showed high concordance with previously defined heterotic groups, correctly assigning 29 out of 30 lines (96.67%) to their expected clusters. In contrast, RNA-seq-derived SNPs showed moderate concordance with expected heterotic groupings (56.67%), indicating that transcriptome-derived markers capture part of the underlying breeding structure, but do not fully resolve heterotic group separation. Overall, these results support the 25k SNP array as a robust tool for assessing genetic relatedness and population structure in maize breeding programs, while RNA-seq-derived SNPs provide complementary but less reliable information for routine heterotic assignment.

## 1. Introduction

Hybrid breeding is the foundation of modern maize (*Zea mays* L.) improvement, relying heavily on the use of genetically divergent parental inbred lines that belong to different heterotic groups. Understanding genetic relationships among inbreds is therefore critical for effective organization of germplasm and the efficient exploitation of heterosis in maize breeding programs [1,2,3]. Prior to the widespread use of molecular markers, inbred line differentiation relied primarily on pedigree information, combining ability tests, and morphological traits [4,5]. While these approaches have long been central to breeding programs, they are limited by factors such as incomplete pedigree records and derivation of new lines from mixed or unknown genetic background/heterotic origin (i.e., open pollinated sources, increased use of proprietary germplasm with expired USA plant variety protection of unknown origin, etc.) [5].

Molecular markers such as Restriction Fragment Length Polymorphisms (RFLPs), Random Amplified Polymorphic DNA (RAPDs), Simple Sequence Repeats (SSRs), Amplified Fragment Length Polymorphisms (AFLPs), and Single Nucleotide Polymorphisms (SNPs) [2,6,7,8,9,10,11] have long provided an objective and efficient means of characterizing genetic diversity and population structure in maize breeding programs. Among these, SNPs have become the markers of choice due to their abundance, genome-wide distribution, and suitability for high-throughput genotyping [3,12,13,14,15,16,17,18]. SNPs can be obtained either through sequencing approaches, such as whole-genome sequencing (WGS) or ribonucleic acid (RNA)-sequencing (RNA-seq), which provide very high marker density for studies like QTL mapping, GWAS, and genomic selection, or through chip-based assays. High-density SNP arrays, such as the Illumina^®^ MaizeSNP50 BeadChip (49,585 loci) and the Affymetrix^®^ MaizeSNP600K (616,201 loci), offer reproducible, cross-comparable, and cost-effective genotyping of large sample sets, making them particularly suitable for evaluating genetic diversity and population structure in breeding collections [3,5,16,17,18,19,20].

Until recently, germplasm classification at the Maize Research Institute “Zemun Polje” (MRIZP) relied primarily on pedigree data, combining ability tests, and, to a lesser extent, SSR markers, which were used to assess genetic relatedness in small sets of maize inbred lines (6–29 lines) or in maize landraces from the genebank (18 accessions), typically employing 10–30 SSR loci [9,10,21,22]. While informative, these approaches were limited in resolution and scalability. Since 2020, MRIZP breeders have adopted a 25k Illumina^®^ Infinum Maize SNP Array (25k SNP array) platform to enhance the precision and efficiency of genetic diversity and population structure analyses in their working collections.

This study was designed to evaluate the reliability and utility of the newly introduced 25k SNP array for routine application in MRIZP’s maize breeding programs. A panel of elite inbred lines with known pedigrees and previously defined heterotic group assignments was selected to test the array. In parallel, total RNA-seq data had been generated for the same set of elite lines for purposes other than genotyping, primarily for functional genomics studies; however, RNA-seq data also contain SNP information that can be extracted and evaluated for genetic analyses. Therefore, SNPs derived from RNA-seq data for this panel were additionally used to assess whether transcriptome-based genotyping could provide comparable insights into genetic relatedness and population structure. The objectives of this study were to: (1) analyze the genetic relatedness and population structure of elite MRIZP maize inbred lines using SNPs from 25k SNP array and RNA-seq, (2) assess the concordance between obtained results and known pedigree/heterotic group information, and (3) compare results obtained from 25k SNP array data with those derived from RNA-seq-based SNPs to evaluate their suitability for breeding applications, primarily for examining genetic relatedness and population structure of maize materials used in MRIZP maize breeding programs.

While it is generally assumed that RNA-seq and SNP arrays differ in their performance for genotyping, this assumption has rarely been tested quantitatively in a breeding-relevant context. In maize, RNA-seq datasets are frequently generated for functional genomics studies and may contain variant information that can be secondarily utilized for genetic analyses. However, clear empirical guidance on whether such RNA-seq-derived SNPs can reliably support population structure inference and heterotic group assignment in elite breeding collections is largely lacking.

This study addresses this gap by systematically comparing 25k SNP array-derived and RNA-seq-derived SNPs for the same set of elite MRIZP maize inbred lines, with the aim of evaluating the scope and limitations of transcriptome-derived SNPs for population genetic inference. The MRIZP core breeding collection represents a historically diverse, program-specific germplasm pool, and evaluating marker informativeness in this context provides practical, evidence-based guidance for breeding decisions.

Previous studies have primarily focused on validating RNA-seq SNP calling accuracy or benchmarking sequencing platforms against SNP arrays. In contrast, the present study evaluates the downstream breeding implications of reusing RNA-seq-derived SNPs for population genetic analyses. RNA-seq datasets are frequently reused for SNP discovery and downstream genetic applications, as they represent a rich source of variant information that can be leveraged for marker development and genotyping [23,24]. In particular, we assess whether RNA-seq-derived SNPs can provide consistent and reliable inference of genetic relatedness and population structure compared to 25k SNP array benchmark, and how potential discrepancies may affect key breeding decisions, including heterotic group assignment, parental selection, and population development.

## 2. Materials and Methods

### 2.1. Plant Material

A panel of 28 elite maize (*Zea mays* L.) inbred lines of diverse heterotic origin, currently used in the maize breeding programs at MRIZP, was selected for this study, which was conducted in 2024 and 2025. Based on available pedigree/origin information, chosen lines were previously grouped into four heterotic groups: nine lines belonged to the Lancaster Sure Crop heterotic group (marked as L-1 to L-9), eight lines to the BSSS (Iowa Stiff Stalk Synthetic) heterotic group (B-1 to B-8), three lines to the Iodent heterotic group (I-1 to I-3), and eight lines with mixed or unknown heterotic backgrounds (derived from commercial hybrids) were categorized as Mixed group (M-1 to M-8) (Table 1). Additionally, two public inbred lines, Mo17 and B73, were included as reference checks, representing the Lancaster and BSSS heterotic groups, respectively. In total, 30 inbred lines were analyzed.

### 2.2. Genotyping

#### 2.2.1. 25k Illumina^®^ Infinum Maize SNP Array (25k SNP Array)

Genotyping was conducted in 2020 using 25k Illumina^®^ Infinum Maize SNP Array by TraitGenetics (SGS Institut Fresenius GmbH, Gatersleben, Germany), following a standardized protocol (“https://sgs-institut-fresenius.de/fileadmin/Media/Health_Nutrition/Trait_Genetics/Downloads/HN-Traitgenetics_Genotyping-22-11.pdf (accessed on 25 April 2026)”). For each genotype, 25 seeds were submitted. After DNA extraction using standard CTAB-based protocol [25] and genotyping, raw SNP data were provided by the service provider.

The 25k SNP array comprises a total of 23,908 SNP loci, including over 14,000 SNPs from the Illumina^®^ MaizeSNP50 BeadChip, approximately 9000 SNPs from the MaizeSNP600K array, and nearly 200 additional SNPs. Of these, 23,147 loci have known physical positions, while 761 are unassigned. The chromosomal distribution of SNPs with known positions is as follows: 2654 (Chr1), 2585 (Chr2), 2718 (Chr3), 2456 (Chr4), 2837 (Chr5), 2017 (Chr6), 2099 (Chr7), 1883 (Chr8), 2072 (Chr9), and 1826 (Chr10).

#### 2.2.2. RNA-Seq

For the purpose of RNA-seq, in 2016, 20 plants per inbred line were grown under controlled conditions in greenhouse (day/night: 16 h/8 h, 25 °C/22 °C, light intensity up to 700 µmol/m^2^/s). At the V4 growth stage (fully developed fourth leaf), samples were collected from the third leaf of three representative plants per genotype.

Total RNA was extracted using Gene Jet RNA Purification kit (Thermo Fisher Scientific, Waltham, MA, USA), pooled for each genotype, and used for cDNA library preparation using Illumina TruSeq Stranded Total RNA LT kit (San Diego, CA, USA) with Ribo-Zero. Pair-end sequencing (2 × 150 bp) was performed in 2016 on the MiSeq Illumina platform using the MiSeq Reagent kit v2 (San Diego, CA, USA). This RNA-seq dataset was used in the study by Banović Đeri et al. [26]. However, in this study, raw transcriptomic data from Banović Đeri et al. [26] were used to identify SNPs from expressed genomic regions.

### 2.3. Data Analysis

#### 2.3.1. 25k SNP Array-Derived SNPs

Raw SNP data from the 25k SNP array were filtered using TASSEL 5.0 [27]. For each SNP, the minor allele frequency (MAF) was calculated directly from nucleotide calls (A, C, G, T), excluding missing or ambiguous values. SNPs were discarded if they had fewer than 30 non-missing genotype calls, more than two alleles, or were heterozygous. Only biallelic SNPs with MAF > 0.05 were retained for further analysis.

Filtered genotypes were converted to numeric format on a per-SNP basis. For each SNP, the two observed alleles were encoded as 0 and 2, while missing or invalid values were set to NA. This encoding was applied independently for each SNP to avoid cross-SNP assumptions.

After filtering, 6610 SNP loci were eliminated, and 17,298 SNP loci were retained. Of these, 8.17% corresponded to markers with undefined genomic position (unknown or position = 0) and were therefore excluded, resulting in a final set of 15,885 SNPs derived from the 25k SNP array used for downstream comparisons. Their chromosomal distribution is shown in Table 2.

#### 2.3.2. RNA-Seq-Based SNP Calling and Filtering

RNA-seq data were processed using a custom bioinformatics pipeline. The pipeline included: quality control (QC) using FastQC; adapter trimming and removal of contaminants and low-quality reads (QC < 30) using Trimmomatic v0.32; filtered reads alignment to the B73 maize reference genome v3.0 using TopHat (insert size 130 bp, standard deviation 50 bp, maximum intron size 100.000); and transcript assembly using Cufflinks v2.2.1 followed by Cuffmerge. SNP calling was performed using BCFtools v1.19, and indels were excluded from further analyses. Additional quality control filters were applied as follows: MAF (minor allele frequency) > 0.05; QUAL score > 20, 20 < DP (read depth) < 200 and SNPs missing rate < 0.5.

After quality filtering, a total of 81,430 SNP loci were retained, including both homozygous and heterozygous sites (ALL dataset). From the initial ALL dataset, two additional datasets were generated: (i) a HOM dataset excluding all heterozygous sites, resulting in 1555 SNPs, and (ii) a FINAL dataset in which heterozygous sites were recoded as missing values (NA) and the data were subsequently re-filtered using the original control criteria, resulting in 22,396 SNPs. Their chromosomal distributions are shown in Table 2.

To evaluate the impact of heterozygous SNP handling in RNA-seq data, these three datasets (ALL, HOM, and FINAL) were used in downstream comparative analyses and subsequently evaluated to identify the most appropriate dataset for comparison with 25k SNP array data.

#### 2.3.3. Cross-Platform SNP Overlap Analysis Between RNA-Seq and 25k SNP Array Datasets

To assess the overlap between RNA-seq-derived and 25k SNP array-derived SNPs, genomic positions were intersected between the three RNA-seq SNP datasets (ALL, HOM, FINAL) and the 25k SNP array dataset. Overlap analysis was conducted using custom Python (v3.13.9) scripts, based on matching chromosomal coordinates/position and classifying matches as direct, reverse-strand, or discordant.

Shared SNPs were further annotated using maize reference genome versions v3, v4, and v5 to determine their genomic context, based on annotations from Ensembl Plants (https://plants.ensembl.org/index.html) and MaizeGDB (https://maizegdb.org/), both accessed on 25 April 2026.

#### 2.3.4. Linkage Disequilibrium (LD) Pruning Sensitivity Analysis and Parameter Selection Using PERMANOVA and Mantel Test

RNA-seq and 25k SNP array HapMap genotype matrices were analyzed in R (v4.3.3) after recoding genotype calls to 0/1/NA (non-ACGT and IUPAC ambiguity codes set to missing). SNPs were retained only if polymorphic and with site-level missingness ≤ 0.5; pairwise IBS-like distances were computed as 1 − mean(g_i = g_j) over jointly observed loci, requiring at least 100 shared loci per sample pair (min_shared = 100). LD thinning was applied separately within each platform by removing markers in high LD (pairwise r^2^ > threshold) within physical windows (100 kb or 500 kb), with an order-based fallback window of 50 SNPs for markers lacking valid coordinates and a minimum of five jointly observed genotypes for r^2^ estimation (min_obs_cor = 5). A grid of LD parameters (r^2^ ∈ {0.1, 0.2, 0.5} × window ∈ {100 kb, 500 kb}) was evaluated against a no-LD baseline using PERMANOVA (adonis2) and Mantel tests (9999 permutations).

Based on marker retention and stability of downstream results, r^2^ = 0.2 with a 500 kb window (max_missing = 0.5) was selected as the primary LD-pruned configuration; downstream analyses therefore compare non-LD versus this selected LD setting.

The selected LD pruning parameters were subsequently used in all downstream analyses, including cross-platform comparisons described in Section 2.3.6, Statistical Analysis and Visualization.

#### 2.3.5. Genetic Distance and Population Structure Analysis

Assessing population structure and genetic relatedness is essential in maize breeding to effectively exploit heterosis [3]. It enables the identification of potential heterotic pairs, the development of new source populations, and the classification of inbred lines into groups and subgroups, particularly those with unknown or admixed genetic backgrounds [2]. In this study, we aimed to determine whether clustering of 30 evaluated inbred lines, based on 25k SNP array and RNA-seq-derived SNP markers, corresponds to their previously defined heterotic groups derived from pedigree information.

Pairwise genetic distance matrices were generated using TASSEL v5.0, and average genetic distances within and between heterotic groups were calculated. Population structure analysis was performed using STRUCTURE v2.3.4 [28,29].

Although the evaluated panel included four breeder-defined origin categories (Lancaster, BSSS, Iodent, and Mixed), population structure analysis was performed under K = 3 because the Mixed group does not represent a discrete heterotic group, but rather a set of lines with admixed or uncertain ancestry derived from multiple breeding sources. Thus, K = 3 was selected as a hypothesis-driven setting corresponding to the three canonical heterotic groups represented in the panel (Lancaster, BSSS, and Iodent).

This choice was formally validated using the DeltaK method described by Evanno et al. [30]. Five independent runs were performed for each K value (ranging from 2 to 7) for both the 25k SNP array and RNA-seq SNPs. To ensure convergence, the burn-in period and Markov Chain Monte Carlo (MCMC) length were increased to 10,000 and 25,000 iterations, respectively. The analysis revealed a distinct peak at K = 3 for both marker datasets (Supplementary Figures S1 and S2), confirming that three clusters represent the optimal population structure, which aligns with the expected pedigree-based grouping.

Thus, population structure was inferred assuming K = 3, and a membership probability threshold of ≥0.7 was used to assign lines to specific groups. This threshold is commonly applied in maize breeding to ensure reliable assignment and effective exploitation of heterosis [3].

All analyses performed on the 25k SNP array dataset were replicated using RNA-seq-derived SNPs. The resulting population structure was compared between the two genotyping platforms and with pedigree-based heterotic group classifications.

#### 2.3.6. Statistical Analysis and Visualization

Principal component analysis (PCA) was used for within-RNA-seq dataset comparisons, whereas principal coordinate analysis (PCoA) was used for cross-platform ordination based on pairwise identity-by-state (IBS)-based distance matrices among 30 inbred lines.

IBS matrices were computed in PLINK v1.9 using --distance square ibs, generating symmetric .mibs outputs. PLINK .mibs outputs were treated as IBS similarity matrices; where distance-based ordination or clustering was required, IBS-based distances were calculated as 1 − IBS.

##### Intra RNA-Seq Dataset Assessment

Comparative analyses were performed across the three RNA-seq SNP datasets (ALL, FINAL, and HOM) to evaluate the impact of heterozygous-site handling on pairwise genetic similarity structure and population structure inference. For each dataset, an IBS similarity matrix was computed. Principal component analysis (PCA) was performed independently for each dataset using --pca 10, producing .eigenvec and .eigenval files. IBS matrices were visualized as heatmaps to compare pairwise similarity patterns among samples, and PCA scatter plots (PC1 vs. PC2) were used to assess changes in clustering across datasets. Summary statistics of off-diagonal IBS values (mean, standard deviation, median, and the 5th–95th percentiles; excluding diagonal self-comparisons) were computed to quantify shifts in pairwise similarity distributions across datasets.

##### Cross-Platform SNP Concordance Analysis Between RNA-Seq and 25k SNP Array Datasets

All analyses were performed using both the non-LD-pruned dataset and the LD-pruned dataset obtained under the selected configuration (r^2^ = 0.2; 500 kb), as defined in Section 2.3.4.

Samples were assigned to four predefined heterotic groups based on pedigree information: BSSS, Lancaster, Iodent, and Mixed. Population differentiation was assessed using PERMANOVA (adonis2, vegan package) with 9999 permutations. Results are reported as sums of squares, degrees of freedom, F-statistics, and the proportion of explained variance (R^2^).

Concordance between FINAL RNA-seq-based and 25k SNP array-based genetic relationship patterns was evaluated using a Mantel test with Pearson correlation and 9999 permutations (implemented in the vegan package).

Principal coordinate analysis (PCoA) based on symmetric IBS-based distance matrices (1 − IBS) was performed for both marker datasets (25k SNP array and FINAL RNA-seq-derived SNPs), and results were visualized using the R (v4.3.3) packages ggplot2 and ggrepel.

Additionally, a joint cross-platform comparison (ΔIBS/Procrustes) was performed using IBS similarity matrices generated in PLINK with --distance square ibs flat-missing. Cross-platform differences were summarized as ΔIBS = IBS_array − IBS_RNA and visualized as ΔIBS heatmaps. Sample configurations were compared via two-dimensional ordination of 1 − IBS dissimilarities (MDS/PCoA) and aligned between platforms using Procrustes analysis; Procrustes disparity (residual sum of squared differences after optimal alignment) was reported.

To further validate the observed genetic relationships, hierarchical clustering was performed using the UPGMA method (average linkage) based on IBS-based distance matrices using the hclust() function in R.

Finally, to specifically assess marker-level overlap between datasets, a custom Python pipeline was developed to identify shared SNP loci, classify concordance patterns (exact match, reverse complement concordance, strand swap, and discordant calls), and quantify overlap between RNA-seq-derived SNP datasets and the 25k SNP array. This analysis complemented PLINK-based distance and population structure analyses by providing locus-level resolution of cross-platform SNP agreement.

## 3. Results

### 3.1. Impact of Heterozygous SNP Handling on RNA-Seq-Based Analyses

Chromosome-level distribution of SNP loci differed between the 25k SNP array and the RNA-seq-derived datasets and shifted modestly across heterozygous-site treatments (Table 2). The 25k SNP array showed a relatively even distribution across chromosomes (6.67–12.02% per chromosome) and included an “Unknown” category (3.51% of loci). In contrast, RNA-seq SNPs were assigned exclusively to the 10 chromosomes and showed greater chromosome-to-chromosome variability, consistent with transcriptome-based ascertainment. Across the RNA-seq datasets, heterozygous-site handling primarily altered the relative representation of loci across chromosomes rather than concentrating SNPs into a small subset of chromosomes, indicating broadly genome-wide coverage despite treatment-dependent retention biases. Relative to ALL, the FINAL dataset retained approximately 19–32% of loci per chromosome, whereas HOM retained only ~1–3%, indicating that most loci in the ALL callset were heterozygous across all chromosomes, with only modest chromosome-specific differences in locus removal.

**Figure 1:**
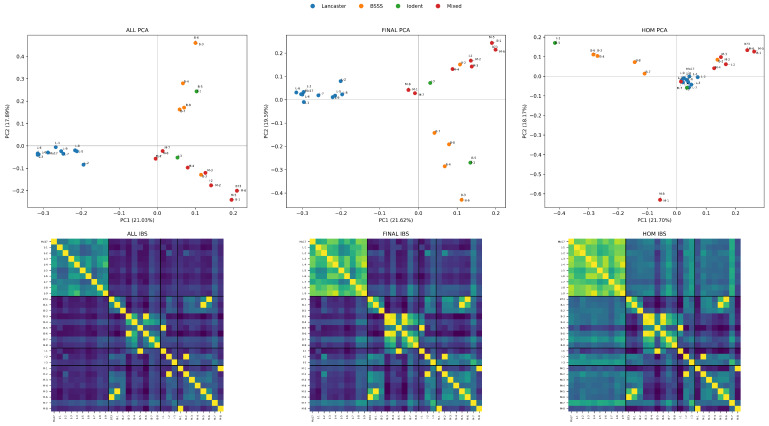
Intra-dataset comparison of RNA-seq SNP datasets (ALL, FINAL, and HOM) under alternative heterozygous-site treatments. Top panel: PCA scatter plots (PC1 vs. PC2) computed separately for each dataset. Bottom panel: pairwise IBS similarity heatmaps. Samples were ordered according to heterotic groups (Lancaster, BSSS, Iodent, and Mixed), with black lines indicating group boundaries. Although PC1 and PC2 explained similar fractions of variance across datasets, sample configurations and clustering patterns differed because principal axes were re-estimated from distinct SNP sets and missing-data profiles. IBS summaries indicate the most compressed similarity structure in ALL, an intermediate and more balanced structure in FINAL, and the broadest dispersion in HOM.

Principal component analysis (PCA) and identity-by-state (IBS)-based pairwise similarity heatmaps revealed pronounced differences in genetic relationship structure among the three RNA-seq SNP datasets (ALL, FINAL, and HOM), reflecting alternative treatments of heterozygous sites (Figure 1). Following heterozygous-site handling, SNP retention differed substantially: FINAL retained 22,396 loci (27.5% of ALL), whereas HOM retained 1555 loci (1.9% of ALL; 6.9% of FINAL). In the FINAL dataset, PCA and IBS were computed on 22,396 variants and 30 samples, with a total genotyping rate of 0.672056 after QC. Across datasets, the fraction of variance captured by the first two principal components was similar (PC1 ≈ 21% and PC2 ≈ 18–20%; ALL: 21.03%/17.89%, FINAL: 21.62%/19.59%, HOM: 21.70%/18.17%); however, sample configurations and clustering patterns differed, consistent with PCA axes being re-estimated independently from different SNP sets and missing-data profiles. In parallel, IBS similarity distributions shifted markedly (*n* = 30; *n*_pairs = 435). The ALL dataset showed the most compressed similarity space, with the highest overall similarity and lowest dispersion (mean IBS = 0.7610; SD = 0.0504; median = 0.7464; 5th–95th = 0.7099–0.8559). The FINAL dataset exhibited lower similarity and a broader dynamic range (mean = 0.6758; SD = 0.1009; median = 0.6451; 5th–95th = 0.5635–0.8969), whereas the HOM dataset showed the widest dispersion and strongest tails (mean = 0.6690; SD = 0.1156; median = 0.6587; 5th–95th = 0.4887–0.8949). Overall, these results indicate that heterozygous-site treatment substantially reshapes IBS-based similarity space and can alter PCA clustering topology, with ALL producing the most compressed similarity structure, HOM the most dispersed and tail-heavy structure, and FINAL representing the most balanced compromise for downstream comparison.

### 3.2. Sensitivity of Results to Linkage Disequilibrium Structure: PERMANOVA and Mantel Test

An LD/missingness sensitivity grid was evaluated at max_missing = 0.5 by varying LD thresholds (r^2^ ∈ {0.1, 0.2, 0.5}) and window sizes (100 kb, 500 kb), alongside a non-LD baseline (Supplementary Table S1). Across the grid, cross-platform distance concordance (Mantel correlation) and within-dataset heterotic-group signal (PERMANOVA) showed stable patterns, whereas marker retention varied with pruning stringency. The intermediate LD setting (r^2^ = 0.2 and 500 kb window) provided the best balance between retaining sufficient markers and maintaining stable downstream patterns and was therefore selected as the primary LD-pruned configuration for subsequent cross-platform comparisons (retaining 4794 RNA-seq SNPs and 5546 25k array SNPs). Overall, LD pruning influenced marker retention more strongly than it altered the qualitative interpretation of genetic relationship patterns and heterotic-group structure.

### 3.3. Genetic Distance Patterns

#### 3.3.1. 25k SNP Array-Based Genetic Distances

Pairwise genetic distances based on 25k SNP array ranged from 0.028 to 0.501 (Supplementary Table S2). To enhance visualization, a red-white-blue color scale was applied, marking the highest 10% of distances (in total 44 of them) in intense red and the lowest 10% (in total 44 of them) in intense blue. The highest 10% of distances were observed between lines from different groups, while the lowest 10% of distances were observed between lines within the groups.

The greatest distance was observed between lines L-3 and B73 (0.501). The third greatest distance (0.499) was recorded between the two public lines chosen as checks in this study, B73 and Mo17, representing the most renowned heterotic pair [31]. Notably, line B73 exhibited high distances (>0.490) with eight out of nine Lancaster lines, including seven highest recorded distances in this study (0.501, 0.500, 0.499, three times 0.498 and 0.497). In contrast, the lowest distance was observed between Lancaster lines L-1 and L-2 (0.028). Low distances were also noted among Iodent lines (0.070 to 0.104).

In this study, average genetic distances between and within heterotic groups were calculated using pairwise distances derived from the 25k SNP array (Table 3).

#### 3.3.2. RNA-Seq-Derived SNPs-Based Genetic Distances

Pairwise genetic distances estimated using RNA-seq SNPs are shown in Supplementary Table S3. A red-white-blue color scale was applied for better visualization as in the previous pairwise distance matrix. Comparison between the results obtained by 25k SNP array and RNA-seq revealed that chromosome 5 had the highest SNP count in 25k SNP array and ALL dataset, whereas the highest SNP count was observed on chromosome 1 in the FINAL dataset and on chromosome 2 in the HOM dataset. In contrast, chromosome 10 had the lowest SNP count in the 25k SNP array, ALL, and FINAL datasets, while chromosome 6 had the lowest count in the HOM dataset (Table 2).

Further comparison revealed that the 25k SNP array method yielded higher overall average distance (0.400) compared to RNA-seq (0.330). The highest distance obtained by RNA-seq was 0.467 (between line B-6 and lines B-1 and M-5), slightly lower than the 0.501 recorded by the 25k SNP array. On the other side, distance 0.000 was recorded six times between lines: B-3 and B-6; B-5 and I-1; I-2 and M-2; B-1 and M-5; B-73 and M-6; M-1 and M-8.

Results showed that 37 out of 44 (84.09%) lowest distances (given in intensive blue) were recorded between lines within the same group (out of which 27 between Lancaster lines), with the remaining 7 recorded between lines from different groups. On the other hand, 37 out of 44 highest distances (given in intensive red) were observed between lines from different groups, with the remaining 7 observed between lines from BSSS group.

The average distances between and within heterotic groups based on RNA-seq SNPs are given in Table 4.

### 3.4. Population Structure Analysis

#### 3.4.1. 25k SNP Array-Based SNPs

The analysis revealed that 29 out of 30 (96.67%) lines were assigned to clusters consistent with their pedigree-based heterotic groups (Table 5). All BSSS and Iodent lines were assigned to their corresponding clusters 2 (with membership probability from 79.2% to 100%) and 3 (membership probability of 100%), respectively. As expected, the lines from Mixed group were not assigned to any of these three clusters, showing majority membership probabilities to clusters 2 and 3 (BSSS and Iodent, respectively). Membership probabilities of Lancaster lines to their cluster 1 ranged from 65.5% to 100%. Among ten Lancaster lines, nine were classified into cluster 1, with line L-5 remaining unassigned (membership probability of 0.655) (Table 5).

Overall, 14 out of 22 evaluated lines from Lancaster, BSSS, and Iodent groups were assigned correctly (consistent with their defined heterotic groups) with membership probabilities above 99%.

#### 3.4.2. RNA-Seq-Derived SNPs

**Figure 2:**
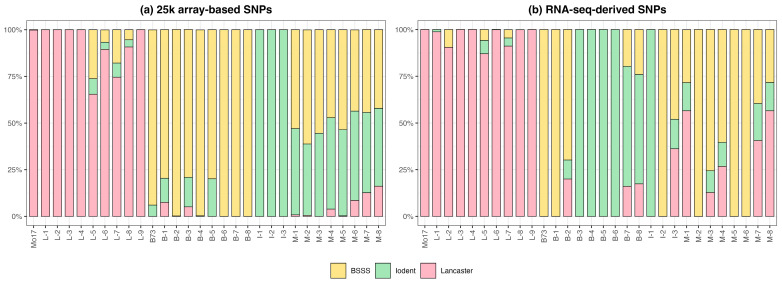
STRUCTURE barplots showing assignment (membership probabilities) of 30 evaluated inbred lines to three assumed clusters (K = 3) for 25k SNP array data (a) and RNA-seq FINAL data (b).

The results obtained from the RNA-seq FINAL dataset differed substantially from those obtained with the 25k SNP array (Figure 2). Concordance with breeder-defined heterotic groups reached 56.67% (Table 5), indicating moderate recovery of the expected heterotic structure, but substantially lower assignment consistency than that observed for the 25k SNP array. In particular, RNA-seq-based clustering did not achieve a clear and stable separation of all heterotic groups, and several lines showed ambiguous placement or overlap among breeder-defined classes. Thus, although RNA-seq-derived SNPs retained biologically meaningful structure, their ability to resolve heterotic grouping remained limited relative to the genome-wide 25k SNP array dataset.

### 3.5. Cross-Platform Comparison of RNA-Seq and 25k SNP Array Genetic Structure

To comprehensively evaluate the concordance between RNA-seq-derived and 25k SNP array-based markers, multiple complementary analytical approaches were applied. These included ordination methods (Principal Coordinate Analysis (PCoA) and Multidimensional Scaling (MDS)/Procrustes), distance-based comparisons (Identity-by-State (IBS) and ΔIBS), clustering analyses (Unweighted Pair Group Method with Arithmetic Mean (UPGMA)), and statistical tests of matrix similarity and group structure (Mantel test and Permutational Multivariate Analysis of Variance (PERMANOVA)), together with graphical assessments through scatter plots and boxplots. Together, these analyses provide an integrated assessment of the extent to which the two marker systems capture comparable patterns of genetic relatedness and population structure.

#### 3.5.1. Principal Coordinate Analysis (PCoA)

**Figure 3:**
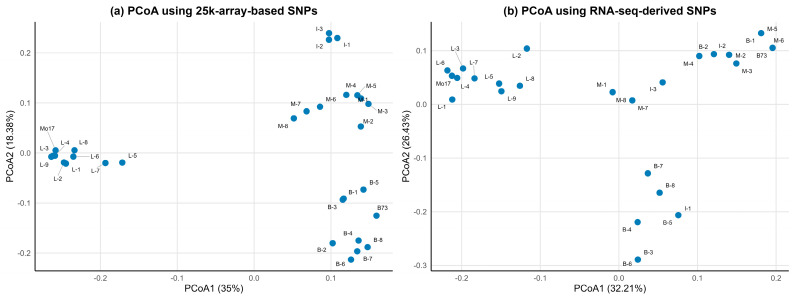
Principal coordinate analysis (PCoA) of 30 evaluated inbred lines based on (a) 25k SNP array-derived SNP data and (b) FINAL RNA-seq-derived SNP data.

Principal coordinate analysis (PCoA) revealed marked differences between marker systems in resolving genetic relationships among the analyzed maize inbred lines (Figure 3). The first two axes explained 53.38% of the total variation for the 25k SNP array (Coordinate 1 = 35.00%, Coordinate 2 = 18.38%) and 58.64% for the RNA-seq SNP dataset (PCo1 = 32.21%, PCo2 = 26.43%).

In the 25k SNP array-based PCoA, the first principal coordinate (PCo1), accounting for 35% of the total genetic variance, clearly differentiated Lancaster from the remaining three groups. Coordinate 2 further differentiated the lines into four groups, consistent with established heterotic patterns: Lancaster lines formed a compact cluster on the left, indicating low within-group variability; Iodent lines clustered tightly in the upper-right quadrant; BSSS lines clustered in the lower-right quadrant; and Mixed lines occupied an intermediate region with greater dispersion along Coordinate 1.

In contrast, the RNA-seq-based PCoA showed weaker and less consistent group separation. Although Lancaster lines remained broadly grouped on the left side of the plot, their dispersion was higher than in the 25k SNP array-based analysis. BSSS and Mixed lines showed partial grouping, but with greater spread across both axes, and Iodent lines did not form a single distinct cluster, overlapping with BSSS and Mixed lines. Several genotypes (including B73 and some Mixed lines) appeared displaced relative to the clearer 25k SNP array-based structure, resulting in less well-defined boundaries among heterotic groups.

These results are consistent with the average within-group distances presented in Table 3 and Table 4 and the pairwise distance matrix results provided in Supplementary Tables S2 and S3.

Taken together, the PCoA results indicate that RNA-seq-derived SNPs recover part of the expected heterotic structure, but with weaker separation, greater dispersion, and more overlap among groups than the 25k SNP array.

#### 3.5.2. Genetic Distance and Population Structure Concordance

**Figure 4:**
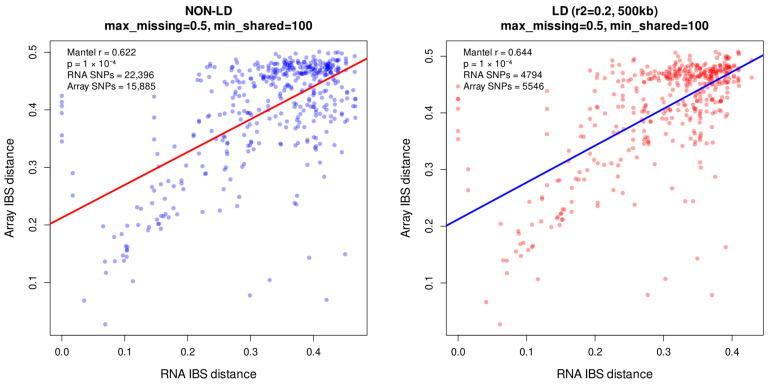
Comparison of pairwise IBS distances derived from RNA-seq and 25k SNP array data before and after LD pruning. Scatterplots showing the association between pairwise IBS distances computed from RNA-seq genotypes (x-axis) and 25k SNP array genotypes (y-axis) for the same sample pairs. The left panel shows the non-LD dataset, while the right panel shows the LD-pruned dataset obtained using an LD threshold of r^2^ = 0.2 within a 500 kb window. Each point represents one pairwise sample comparison, and the solid line indicates the linear regression fit. Mantel correlation coefficients and *p*-values are provided in each panel, demonstrating significant concordance between the two marker datasets.

Pairwise distances from RNA-seq and 25k SNP array datasets were positively associated in both settings (Figure 4). The Mantel correlation increased slightly after LD pruning (non-LD: r ≈ 0.622; LD-pruned: r ≈ 0.644; *p* = 1 × 10^−4^ in both), indicating broadly consistent cross-platform distance structure with modest improvement after LD thinning.

**Figure 5:**
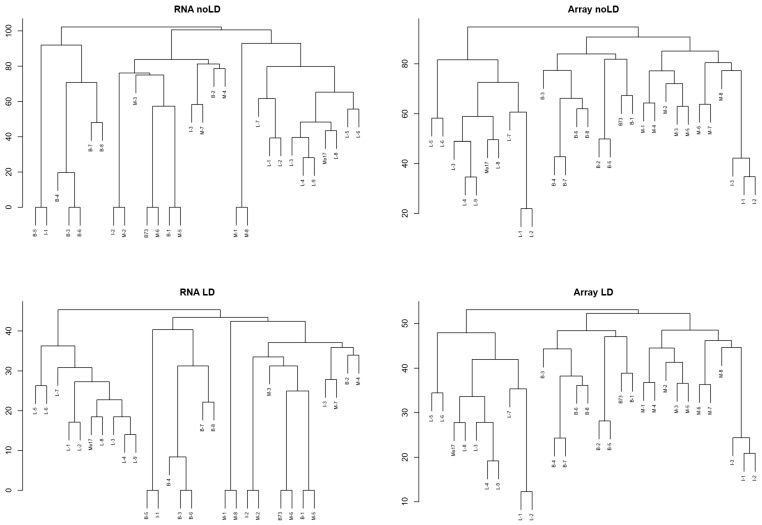
UPGMA clustering of inbred lines from IBS-based distances: 25k SNP array vs. RNA-seq, non-LD vs. LD-pruned. UPGMA trees were constructed separately from 25k SNP array (left column) and RNA-seq (right column) IBS-like distance matrices. Top row: non-LD. Bottom row: LD-pruned setting (r^2^ = 0.2; 500 kb; max_missing = 0.5). Labels correspond to maize inbred lines; branch lengths reflect IBS-based distance.

UPGMA clustering (Figure 5) revealed broadly comparable grouping patterns between non-LD and LD-pruned conditions within each platform. LD pruning produced only modest local rearrangements while preserving the main clustering structure, supporting stability of the inferred relationships under LD thinning.

**Figure 6:**
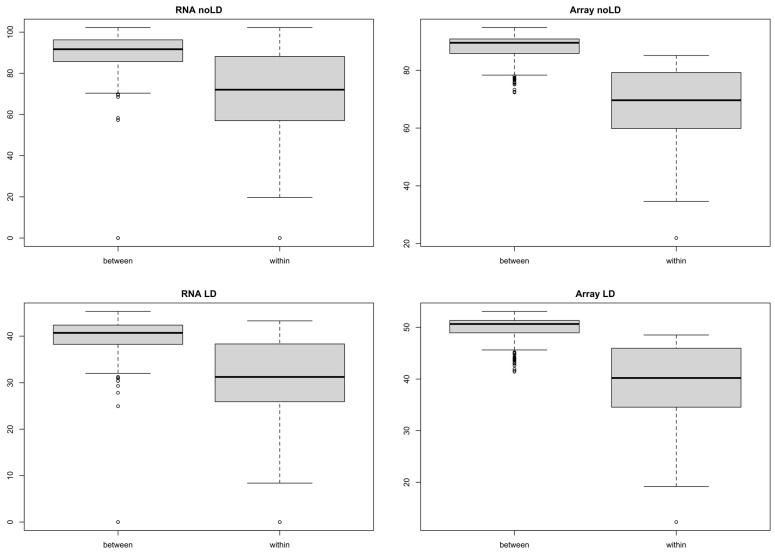
Within- vs. between-group distance separation for 25k SNP array and RNA-seq, with and without LD pruning (max_missing = 0.5). Boxplots summarize distributions of pairwise IBS-based distances for comparisons within heterotic groups versus between heterotic groups, shown separately for the 25k SNP array (left) and RNA-seq (right). Top row: non-LD. Bottom row: LD-pruned setting (r^2^ = 0.2; 500 kb). Boxes show median and interquartile range; whiskers indicate dispersion; points represent outliers.

In both platforms and under both conditions, between-group distances were higher than within-group distances (Figure 6), indicating detectable heterotic-group structure. LD pruning reduced the overall spread of distances while preserving the within < between ordering, consistent with heterotic differentiation being retained after LD thinning.

Together, scatter/Mantel concordance, UPGMA topology, and within–between distance separation show that LD pruning has a modest quantitative effect, but does not materially change the qualitative interpretation of cross-platform genetic distance structure.

#### 3.5.3. Ordination-Based Comparison (MDS/PCoA) and ΔIBS

**Figure 7:**
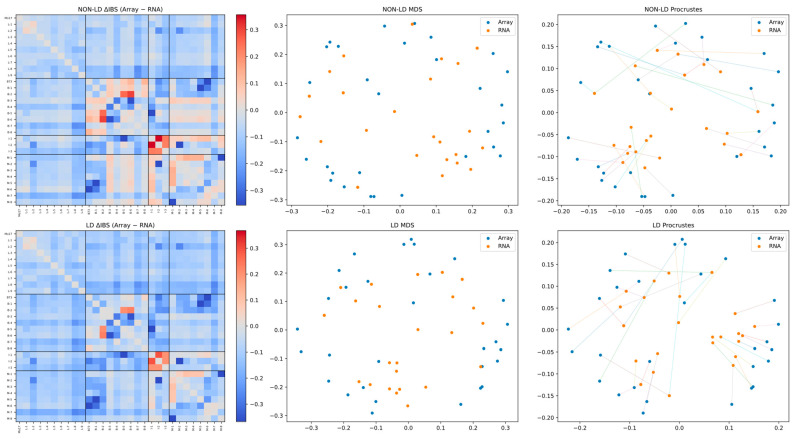
Comparative analysis of RNA-seq and 25k SNP array IBS similarity structure under NON-LD and LD-pruned SNP datasets using ΔIBS heatmaps, MDS ordination, and Procrustes analysis. Rows correspond to analyses performed using the complete NON-LD SNP dataset (top) and the LD-pruned SNP dataset (r^2^ = 0.2, 500 kb window; bottom). Left panels show heatmaps of pairwise IBS similarity differences (ΔIBS = 25k SNP array—FINAL RNA), where positive values indicate higher similarity estimates in 25k SNP array data and negative values indicate higher similarity estimates in the RNA-seq-derived data. Samples were ordered according to heterotic groups (Lancaster, BSSS, Iodent, and Mixed), with black lines indicating group boundaries. Middle panels display two-dimensional multidimensional scaling (MDS) projections generated from IBS-derived genetic distance matrices (1−IBS) for 25k SNP array and FINAL RNA datasets. Right panels show Procrustes superimposition analyses comparing MDS configurations derived from the 25k SNP array and RNA-seq datasets. Lines connect corresponding samples between datasets, with shorter distances indicating stronger concordance between ordinations. Procrustes disparity values quantify the overall similarity between RNA-seq-derived and 25k SNP array-derived genetic structures.

In the unpruned comparison, ΔIBS (IBS_array − IBS_RNA) was shifted below zero (median ΔIBS = −0.0711; 5th–95th percentile = −0.2023 to 0.0574), and 6.44% of sample pairs showed |ΔIBS| > 0.2. Overall correspondence between off-diagonal IBS values was moderate (Spearman ρ = 0.4768, *p* = 4.48 × 10^−26^). Six off-diagonal sample pairs reached IBS_RNA = 1.0 (B73–M-6, B-1–M-5, B-3–B-6, B-5–I-1, I-2–M-2, M-1–M-8), yielding the largest negative ΔIBS values (maximum |ΔIBS| = 0.4244). Procrustes-aligned ordinations based on 1−IBS distances showed substantial residual mismatch between platforms (Procrustes disparity = 0.5583) (Figure 7).

Under the LD-pruned setting (r^2^ = 0.2; 500 kb), 4794 RNA-seq SNPs and 5546 array SNPs were retained. ΔIBS shifted further below zero (median ΔIBS = −0.1009; 5th–95th percentile = −0.2309 to −0.0046), and 8.97% of pairs had |ΔIBS| > 0.2. Concordance between IBS matrices increased (Spearman ρ = 0.5435, *p* = 8.53 × 10^−35^), while Procrustes mismatch remained similar (disparity = 0.5576). The same six IBS_RNA = 1.0 pairs persisted after LD pruning (Figure 7).

Thus, LD pruning increased matrix-level concordance (higher Spearman ρ) but did not reduce the Procrustes mismatch, and the same IBS_RNA = 1.0 pairs persisted, indicating that the main cross-platform patterns were robust to LD pruning.

### 3.6. Cross-Platform SNP Overlap Between RNA-Seq and 25k SNP Array Datasets

A very limited overlap was observed between RNA-seq-derived SNPs and 25k SNP array markers. Specifically, six shared SNP positions were identified between the ALL RNA-seq dataset and the 25k SNP array dataset, while only two shared positions were retained in the FINAL dataset. No overlapping SNPs were detected between the HOM dataset and the 25k SNP array.

A detailed examination of the overlapping SNP positions revealed that among the six shared loci between the RNA-seq (ALL) and 25k SNP array datasets, two represented exact allele matches, two showed reverse complement concordance (strand-consistent matches with allele reversal), while the remaining two exhibited discordant genotype calls. In the FINAL RNA-seq dataset, two overlapping SNPs were retained, corresponding to one reverse complement swap and one strand swap.

Functional annotation of the six shared SNPs (including the two retained in the FINAL dataset), based on maize genome annotations (v3, v4, and v5), revealed that all variants were located within coding sequences (CDS/exonic regions) of genes (Table 6).

Across the six overlapping loci identified in ALL, concordance categories included one direct match (CHR5:2795466; Transcription factor IWS1/SPN1), one allele swap (CHR9:314644; potassium outward rectifying channel), two strand/orientation-related matches consistent with reverse-complement relationships (CHR2:136193; EXO70A1; and CHR2:214661090; CYP727A4/DUF), and two mismatches (CHR2:232838881; ZmMTP1-1; and CHR9:20238182; Transcription factor mads67). After heterozygote recoding and re-filtering (FINAL), only two of the four concordant loci remained shared with the 25k SNP array (CHR2:214661090 and CHR9:314644), whereas the other two concordant loci (CHR2:136193 and CHR5:2795466) were lost during recoding/re-filtering.

Overall, cross-platform analyses indicate that RNA-seq-derived SNPs retain moderate concordance with the broader genetic relationship patterns captured by the 25k SNP array, but they provide weaker, less stable, and less clearly resolved heterotic-group structure.

## 4. Discussion

This study evaluates the performance of RNA-seq-derived SNPs relative to a 25k SNP array for estimating genetic relationships, population structure, and heterotic grouping in elite maize breeding germplasm. While RNA-seq-derived SNPs and SNP array-based genotypes are generally considered to differ in their suitability for genetic distance estimation and population structure analysis, this relationship has rarely been quantitatively assessed in breeding-relevant contexts [32]. In maize, RNA-seq datasets are increasingly reused for secondary genomic analyses due to their availability and cost efficiency [23]. The analysis therefore focuses on whether transcriptome-derived variants can provide comparable breeding-relevant information to genome-wide SNP markers when applied to the same genetic panel and evaluated using complementary distance-, ordination-, and clustering-based approaches.

The results consistently show that while both marker systems capture broad patterns of genetic variation, they differ in resolution and stability of inferred structure, particularly in the context of heterotic group assignment. These differences are further reflected across distance-based concordance metrics, ordination patterns, and cross-platform similarity measures, highlighting the influence of marker ascertainment and genomic representation on downstream genetic inference.

### 4.1. Technical Considerations in SNP Genotyping: RNA-Seq vs. 25k SNP Array (Missingness, Marker Distribution, and Site Filtering)

RNA-seq-derived SNP datasets have technical characteristics that can influence genetic distance estimation and population structure inference. RNA-seq variant discovery is restricted to expressed regions (at a specific time in a specific tissue) and is affected by heterogeneous transcript abundance, uneven read coverage, and allele-specific effects, which can introduce dataset-specific biases in genotype representation [24,33,34]. On the other hand, SNPs derived from DNA SNP arrays for maize represent genome-wide, stable genetic variation and are independent of tissue type (e.g., root vs. leaf), because the genotype is identical across all tissues. Thus, SNP arrays provide the genome-wide coverage and consistency necessary to successfully characterize population structure and genetic diversity in diverse maize germplasm panels [3,5], even when comparing regional gene bank panels across different continents [20].

Read mapping rates indicated generally high alignment efficiency to the reference genome, with no evidence of systematic mapping bias across heterotic groups, but genotype completeness varied substantially among samples, consistent with heterogeneous transcriptomic coverage across samples inherent to RNA-seq SNP discovery. Per-sample missingness ranged from 13.39 to 63.85% in ALL, 15.88 to 62.64% in HOM, and 16.15 to 67.33% in FINAL (Supplementary Table S4). In the FINAL dataset, this elevated missingness is consistent with the heterozygote-to-missing recoding and subsequent re-filtering and is also reflected in PLINK’s total genotyping rate after QC (0.672056). Consequently, call rate is driven by transcript abundance and sequencing depth rather than genomic presence, and the absence of clear heterotic-group-specific missingness patterns suggests that genotype incompleteness is predominantly technical rather than structure-driven.

Consistent with transcriptome-based ascertainment, SNP representation across chromosomes was non-uniform, reflecting enrichment toward gene-rich and actively transcribed regions rather than uniform genome-wide coverage. Chromosome-level marker densities ranged from 2.08 to 6.18 SNP/Mb in ALL, 0.67 to 1.59 SNP/Mb in FINAL, and 0.05 to 0.10 SNP/Mb in HOM (Table 2). These shifts are expected when variant discovery depends on expression and coverage, and they provide important context for interpreting distance- and structure-based results derived from RNA-seq SNPs.

Because RNA-seq SNPs cluster in expressed loci that may be locally correlated, linkage disequilibrium (LD) can, in principle, disproportionately influence distance-based analyses when clusters of highly correlated markers dominate the signal. We therefore evaluated a range of LD pruning thresholds and window sizes relative to a non-pruned baseline. Across these settings, cross-platform concordance and heterotic-group differentiation remained broadly stable. Based on marker retention and downstream consistency, the r^2^ = 0.2 and 500 kb configuration was used as the primary LD-pruned setting; however, comparisons with the non-pruned dataset showed that LD pruning did not materially change the main biological interpretation (Supplementary Table S1).

Given the combined effects of missingness heterogeneity and non-uniform genomic representation in RNA-seq SNPs, we assessed how alternative treatments of heterozygous calls reshape locus retention and the resulting IBS/PCA similarity structure within the RNA-seq datasets. In practice, heterozygous-site handling produced large differences in effective information content: FINAL retained 22,396 SNPs (27.5% of ALL), whereas HOM retained 1555 SNPs (1.9% of ALL; 6.9% of FINAL), and FINAL exhibited increased missingness consistent with heterozygote-to-missing recoding. Chromosome-level retention was similarly non-uniform (FINAL ~19–32% of ALL loci per chromosome; HOM ~1–3%), indicating that heterozygous calls dominated the unfiltered RNA-seq callset genome-wide (using the ALL→HOM reduction as a proxy). These preprocessing differences translated into distinct IBS outcomes: ALL showed the most compressed similarity distribution (mean IBS = 0.7610; SD = 0.0504; 5th–95th = 0.7099–0.8559), FINAL exhibited lower central tendency and a broader dynamic range (mean = 0.6758; SD = 0.1009; 5th–95th = 0.5635–0.8969), and HOM showed the broadest dispersion with stronger tails (mean = 0.6690; SD = 0.1156; 5th–95th = 0.4887–0.8949). Since PLINK’s IBS output is a similarity measure and explicitly accounts for missing calls, shifts in missingness and locus availability can directly alter the effective information contributing to pairwise similarity estimates. Although PC1/PC2 variance fractions were similar across datasets, clustering topology differed because PCA axes are re-estimated independently from different SNP sets and missing-data profiles; therefore, the FINAL dataset was selected as a pragmatic compromise for downstream comparisons with the 25k SNP array data.

In contrast to RNA-seq-specific challenges, the 25k SNP array dataset required relatively minimal preprocessing. Filtering was primarily limited to standard quality control steps, including the removal of SNPs with low call rates, multiallelic variants, heterozygous calls, and low minor allele frequency (MAF ≤ 0.05). In addition, approximately 8.17% of markers lacked defined genomic positions and were excluded due to their incompatibility with downstream analyses. Overall, these filtering steps were straightforward and had a limited impact on marker distribution compared to the more complex biases observed in RNA-seq-derived genotypes.

### 4.2. Cross-Platform Comparison Between RNA-Seq and 25k SNP Array Data

Across ordination-, distance-, and similarity-based analyses, the RNA-seq FINAL and 25k SNP array datasets showed partially concordant patterns of genetic relationships, together with clear platform-specific differences in the representation of population structure and similarity-space geometry. Although the RNA-seq dataset explained a slightly higher proportion of total variation along the first two PCoA axes (58.64% versus 53.38% for the 25k SNP array), this did not translate into improved resolution of heterotic structure. In contrast, the SNP array produced clearer and biologically more interpretable clustering patterns consistent with known heterotic groupings and pedigree expectations, including compact Lancaster and Iodent clusters and clearer separation from BSSS and Mixed lines. RNA-seq-based ordinations showed greater overlap among Iodent, BSSS, and Mixed groups, and less stable placement of several genotypes. These differences likely reflect inherent limitations of RNA-seq-derived SNP ascertainment, consistently with RNA-seq-specific ascertainment effects described in Section 4.1.

Despite these limitations, RNA-seq-derived SNPs retained a biologically meaningful component of the underlying relationship structure. Matrix-level comparisons showed moderate and statistically significant concordance between RNA-seq and array-derived genetic distances in both settings (*p* = 1 × 10^−4^), indicating that broad gradients of relatedness were preserved across platforms. Consistent with this, scatterplot comparisons demonstrated stable positive associations between RNA-derived and array-derived distance spaces under both non-LD and LD-pruned conditions. Likewise, UPGMA dendrograms showed broadly stable within-platform topologies after LD pruning, with only modest local rearrangements, while within-versus-between group distance distributions remained consistent with heterotic-group differentiation in both datasets. Together, these observations indicate that LD pruning produced only modest quantitative changes and did not substantially alter the qualitative interpretation of cross-platform relationship patterns.

However, additional analyses revealed persistent platform-specific differences in similarity-space geometry. ΔIBS values (IBS_array − IBS_RNA) were shifted below zero in both settings (median −0.0711 without LD; −0.1009 with LD), indicating that RNA-seq FINAL generally produced higher pairwise similarity estimates than the SNP array for many genotype pairs. Procrustes analyses likewise showed substantial residual mismatch between array- and RNA-derived ordination spaces in both conditions (disparity ≈ 0.558), demonstrating that the overall sample configurations remained markedly different even after LD pruning. Importantly, the same six off-diagonal genotype pairs reached IBS_RNA = 1.0 in both the unpruned and LD-pruned datasets, indicating that these “collapsed” RNA-based relationships were not solely attributable to LD structure. Because IBS similarity estimates were derived from PLINK .mibs outputs under flat-missing assumptions, these discrepancies most likely reflect platform-dependent differences in effective information content and SNP ascertainment rather than direct marker overlap.

Overall, RNA-seq-derived SNPs showed reduced resolution compared to the 25k SNP array, despite retaining broad signals of genetic relatedness.

### 4.3. Evaluation of SNP Concordance Between RNA-Seq and 25k SNP Array Datasets

The limited overlap between RNA-seq-derived and 25k SNP array markers reflects fundamental differences in variant ascertainment rather than inconsistencies in data quality. SNP arrays target pre-selected genome-wide polymorphic loci, whereas RNA-seq SNP discovery is restricted to expressed regions and depends on transcript abundance, sequencing depth, and gene expression patterns. Consequently, the two platforms interrogate largely non-overlapping portions of the maize genome, resulting in minimal direct SNP-to-SNP concordance.

In the initial RNA-seq callset including both homozygous and heterozygous sites (ALL), six loci overlapped the 25k SNP array by chromosomal position (Table 6). Concordance among these loci included one direct allele match, strand/orientation-related matches, one allele swap, and two mismatches, indicating that cross-platform agreement was limited not only by low positional overlap, but also by differences in allele representation and orientation conventions.

After heterozygous genotypes were recoded as missing values and the dataset was re-filtered (FINAL), only two loci remained shared with the SNP array: chr2:214661090 (PZE-102171277; CYP727A4/DUF) and chr9:314644 (ZmSYNBREED_66628_477; potassium outward rectifying channel) (Table 6). No overlapping loci remained in the homozygous-only dataset (HOM), demonstrating that heterozygous-site handling and downstream filtering substantially reduce the variant space available for direct cross-platform comparison. This reduction likely reflects increased locus-level missingness causing some sites to fail call-rate or allele-frequency filters.

The extremely limited SNP-level overlap indicates that direct marker-to-marker concordance is not an appropriate benchmark for evaluating cross-platform agreement in this study. Instead, comparisons are more informative at the level of genetic distances and population structure, where both datasets retained biologically meaningful relationship signals despite clear differences in heterotic-group resolution. The two loci retained after FINAL filtering may nevertheless represent useful high-confidence cross-platform “anchor” markers for future validation and allele tracking, provided that allele orientation is carefully harmonized prior to interpretation.

### 4.4. Implications for Breeding and Pedigree Inference

Accurate assessment of genetic relatedness and population structure is essential for effective exploitation of heterosis in maize breeding programs. In this study, 25k SNP array-based genotyping consistently showed high concordance with known pedigree information and predefined heterotic groups, confirming its suitability as a reliable tool for routine breeding applications.

In contrast, RNA-seq-derived SNPs were more sensitive to data processing choices, particularly in the treatment of heterozygous sites. Although certain preprocessing strategies, such as the FINAL dataset approach, improved clustering stability, the overall resolution of genetic relationships remained lower than that achieved with the 25k SNP array. This reduced performance is likely driven by uneven genomic representation, higher missingness, and expression-dependent biases inherent to RNA-seq-based variant discovery.

Despite these limitations, RNA-seq-derived SNPs captured broad patterns of genetic relatedness and partially recovered population structure, indicating the presence of biologically meaningful signal. This suggests that RNA-seq-derived SNPs may provide transcript-linked information relevant for functional studies. However, for applications requiring high accuracy and reproducibility—such as heterotic group assignment, pedigree validation, and parental line selection—the 25k SNP array remains the preferred approach due to its standardized marker set, uniform genome coverage, and robustness to technical variation. Beyond method comparison, this study provides material-specific validation of the 25k SNP array for the MRIZP breeding program. Given the historical diversity and partial distinctiveness of MRIZP germplasm relative to commonly used reference panels, this validation is particularly relevant. The results demonstrate that the array reliably captures expected genetic relationships and heterotic structure within this material.

This validation has direct practical implications for breeding decisions at MRIZP. The 25k SNP array can be confidently applied for routine heterotic group assignment of new inbred lines, early-stage parental selection, and identification of genetically divergent crosses prior to field evaluation. This enables more informed crossing decisions and reduces reliance on extensive phenotypic testing at early breeding stages, thereby improving breeding efficiency.

Overall, the contribution of this study lies in defining the limits of method transferability and quantifying their impact on breeding-relevant inference. While RNA-seq-derived SNPs may complement array-based data in functional or gene-centric analyses, genome-wide SNP arrays remain the more reliable tool for assessing genetic relatedness, heterotic grouping, and population structure in applied maize breeding. Although integrating both approaches may enhance breeding precision, the present study is method-oriented and based on a defined set of elite inbred lines; thus, its conclusions are most directly applicable to structured breeding germplasm rather than broad population-genetic contexts.

### 4.5. Practical Considerations

Practical considerations further reinforce the advantages of array-based genotyping for population genetic analyses. In addition to methodological robustness, RNA-seq-based SNP discovery requires substantial bioinformatic expertise and computational infrastructure, which may limit its routine application in breeding programs. In contrast, SNP arrays are widely integrated into breeding pipelines due to their standardized workflows, reproducibility, and minimal computational requirements.

Estimated costs and timelines suggest that the 25k SNP array is on the order of ~EUR 30 per sample with a turnaround time of 4–8 weeks, whereas RNA-seq-based approaches may range from EUR 200 to EUR 400 per sample and require approximately 6–10 weeks, depending on experimental and analytical conditions.

Combined with the technical limitations observed in this study, including high missingness and uneven genomic representation, these factors further restrict the utility of RNA-seq-derived SNP datasets for genome-wide genetic inference.

### 4.6. Study Limitations

While the panel of 30 inbred lines is modest in size, it was intentionally selected to represent the core MRIZP breeding germplasm with well-documented pedigrees and established heterotic group assignments. This design is appropriate for method-oriented, within-panel comparisons of genotyping platforms, but a larger and more diverse panel would be required to capture rare alleles, weak substructure, or fine-scale diversity patterns. Therefore, the present findings should be interpreted as program-specific rather than population-level inferences.

A further limitation of RNA-seq-based genotyping is potential reference bias introduced by alignment to a single linear reference genome. Because reads were mapped to the B73 reference genome, allele recovery may have been favored for haplotypes more similar to B73, potentially reducing the representation of alternative alleles in more divergent genetic backgrounds. This bias may have contributed to platform-specific differences in inferred similarity patterns and heterotic structure.

Finally, direct SNP-to-SNP concordance between RNA-seq-derived and array-derived marker sets is constrained by the minimal positional overlap of loci interrogated by the two platforms, limiting one-to-one validation and motivating reliance on distance- and structure-level comparisons. Distance-matrix comparisons and permutation-based significance tests (e.g., Mantel, PERMANOVA) summarize relationships at the matrix level and are informative for method comparison, but they do not substitute for per-locus concordance assessment when overlap is sparse.

### 4.7. Future Perspectives

Future studies should evaluate whether multi-reference or pangenome-based alignment strategies can reduce reference bias and improve the consistency of RNA-seq-derived genotype inference across diverse heterotic backgrounds. In addition, advances in variant-calling methodologies, including haplotype-aware approaches and improved modeling of allele-specific expression, could further enhance the accuracy of genotype inference from transcriptomic data, particularly for heterozygous sites.

Validation in a larger and more diverse breeding panel, including lines with known heterotic assignment and practical crossing relevance, would help determine how broadly the observed platform differences apply across elite germplasm. Additional work is also needed to develop and benchmark analysis frameworks that more explicitly accommodate admixed breeding materials, since lines of mixed origin may be only partially captured by fixed-cluster assignment schemes such as K = 3.

Further investigation of the functional relevance of RNA-seq-derived SNPs, particularly those affecting coding sequences, transcript isoforms, and gene expression regulation, may provide additional insights into the relationship between genetic variation and gene expression, with potential implications for trait-associated studies in maize breeding.

From an applied breeding perspective, future research should also assess how transcriptome-derived SNP data might best be integrated with routine genomic tools, for example as a complementary source of information in early germplasm characterization or in studies combining genetic structure with gene expression profiles.

## 5. Conclusions

This study demonstrates that genome-wide 25k SNP array data provide robust and biologically interpretable inference of genetic relatedness and heterotic structure in elite MRIZP maize inbred lines. In contrast, RNA-seq-derived SNPs showed moderate concordance with expected heterotic groupings and did not achieve equally clear or stable separation among heterotic groups, indicating that transcriptome-derived markers capture only part of the breeding-relevant population structure.

At the same time, RNA-seq-based SNP data retained meaningful genetic signal and showed significant cross-platform agreement at the level of broader relationship patterns, supporting their value as a complementary source of information. However, because RNA-seq genotype inference is more sensitive to preprocessing choices, transcriptome-dependent marker representation, and potential reference bias, it is less suitable than genome-wide SNP arrays for routine heterotic assignment, pedigree inference, and other breeding decisions requiring high reproducibility and interpretability.

For maize breeding programs, these findings support the 25k SNP array as the preferred platform for practical germplasm classification and heterotic group assessment, while RNA-seq-derived SNPs may be most useful in integrative settings where transcriptomic and genetic information are considered jointly.

## Figures and Tables

**Table 1:** The 30 evaluated inbred lines, their labels, and their pedigree-based heterotic groups.

Number	Line	Origin	Number	Line	Origin	Number	Line	Origin
1	Mo17	Lancaster	11	B73	BSSS	21	I-2	Iodent
2	L-1	Lancaster	12	B-1	BSSS	22	I-3	Iodent
3	L-2	Lancaster	13	B-2	BSSS	23	M-1	Mixed
4	L-3	Lancaster	14	B-3	BSSS	24	M-2	Mixed
5	L-4	Lancaster	15	B-4	BSSS	25	M-3	Mixed
6	L-5	Lancaster	16	B-5	BSSS	26	M-4	Mixed
7	L-6	Lancaster	17	B-6	BSSS	27	M-5	Mixed
8	L-7	Lancaster	18	B-7	BSSS	28	M-6	Mixed
9	L-8	Lancaster	19	B-8	BSSS	29	M-7	Mixed
10	L-9	Lancaster	20	I-1	Iodent	30	M-8	Mixed

**Table 2:** Distribution of analyzed SNP loci across chromosomes in the 25k SNP array and the three RNA-seq datasets (ALL, HOM, and FINAL).

Chromosome	25k SNP Array Loci Before/After Removing Unknown and 0 Positions	% of Total Loci	RNA-Seq Loci (ALL/HOM/FINAL)	% of Total Loci (ALL/HOM/ FINAL)	SNP Density—Number of SNPs per Mb (ALL/HOM/ FINAL)
1	1818/1726	10.51/10.87	11,266/211/3649	13.83/13.57/16.29	4.90/0.09/1.59
2	1989/1892	11.50/11.91	8966/236/2754	11.01/15.18/12.30	3.90/0.10/1.20
3	1959/1850	11.33/11.65	7513/144/2309	9.23/9.26/10.31	3.27/0.06/1.00
4	1880/1801	10.87/11.34	9226/164/2069	11.33/10.55/9.24	4.01/0.07/0.90
5	2079/1980	12.02/12.46	14,223/167/2752	17.47/10.74/12.29	6.18/0.07/1.20
6	1499/1423	8.67/8.96	7942/116/1975	9.75/7.46/8.82	3.45/0.05/0.86
7	1541/1468	8.91/9.24	5828/133/1735	7.16/8.55/7.75	2.53/0.06/0.75
8	1336/1278	7.72/8.04	6326/117/1891	7.77/7.52/8.44	2.75/0.05/0.82
9	1436/1379	8.30/8.68	5365/140/1725	6.59/9.00/7.70	2.33/0.06/0.75
10	1153/1088	6.67/6.85	4775/127/1537	5.86/8.17/6.86	2.08/0.06/0.67
Unknown	608/0	3.51/0	0	0.00	/
Total	17,298/15,885	100.00	81,430/1555/22,396	100.00	35.40/0.68/9.74

**Table 3:** Average distances (minimum–maximum) between (not bolded) and within (bolded) heterotic groups for 25k SNP array-based SNPs.

Heterotic Group	Lancaster Sure Crop	BSSS	Iodent	Mixed
Lancaster Sure Crop	**0.221 (0.028–0.383)**	0.475 (0.429–0.501)	0.465 (0.449–0.486)	0.462 (0.427–0.483)
BSSS		**0.310 (0.102–0.406)**	0.452 (0.385–0.475)	0.408 (0.343–0.456)
Iodent			**0.084 (0.070–0.104)**	0.336 (0.300–0.362)
Mixed				**0.342 (0.225–0.415)**

**Table 4:** Average distances (minimum–maximum) between (not bolded) and within (bolded) heterotic groups for RNA-seq-derived SNPs.

Heterotic Group	Lancaster Sure Crop	BSSS	Iodent	Mixed
Lancaster Sure Crop	**0.147 (0.066–0.284)**	0.386 (0.231–0.466)	0.372 (0.236–0.466)	0.367 (0.216–0.452)
BSSS		**0.297 (0.000–0.467)**	0.325 (0.000–0.433)	0.349 (0.000–0.467)
Iodent			**0.350 (0.299–0.421)**	0.308 (0.000–0.433)
Mixed				**0.301 (0.000–0.417)**

**Table 5:** Assignment (membership probabilities) of 30 evaluated inbred lines to three assumed clusters and accordance of clustering with previously defined heterotic groups for 25k SNP array data (a) and RNA-seq data (b). Numbers marked with green or red show whether the line is clustered (yes or no, respectively) to its corresponding cluster/heterotic group above chosen threshold of 0.7 (70%).

	(a) 25k SNP Array-Based SNPs					(b) RNA-seq-Derived SNPs			
Inbred Line	CLUSTER 1 Lancaster	CLUSTER 2 BSSS	CLUSTER 3 Iodent	Accordance with Defined Heterotic Groups	Inbred Line	CLUSTER 1 Lancaster	CLUSTER 2 BSSS	CLUSTER 3 Iodent	Accordance with Defined Heterotic Groups
Mo17	0.998	0	0.002	YES	Mo17	1	0	0	YES
L-1	1	0	0	YES	L-1	0.988	0	0.012	YES
L-2	1	0	0	YES	L-2	0.905	0.095	0	YES
L-3	1	0	0	YES	L-3	1	0	0	YES
L-4	1	0	0	YES	L-4	1	0	0	YES
L-5	0.655	0.262	0.083	NO	L-5	0.871	0.058	0.071	YES
L-6	0.895	0.066	0.038	YES	L-6	0.999	0	0.001	YES
L-7	0.746	0.179	0.075	YES	L-7	0.912	0.044	0.043	YES
L-8	0.908	0.054	0.038	YES	L-8	0.999	0	0	YES
L-9	1	0	0	YES	L-9	1	0	0	YES
B73	0	0.939	0.06	YES	B73	0	1	0	YES
B-1	0.075	0.796	0.129	YES	B-1	0	1	0	YES
B-2	0.001	0.999	0.001	YES	B-2	0.2	0.698	0.102	NO
B-3	0.051	0.792	0.158	YES	B-3	0	0	1	NO
B-4	0	0.996	0.003	YES	B-4	0	0	1	NO
B-5	0	0.799	0.201	YES	B-5	0	0	1	NO
B-6	0	1	0	YES	B-6	0	0	1	NO
B-7	0	1	0	YES	B-7	0.161	0.197	0.642	NO
B-8	0	1	0	YES	B-8	0.174	0.24	0.586	NO
I-1	0	0	1	YES	I-1	0	0	1	YES
I-2	0	0	1	YES	I-2	0	1	0	NO
I-3	0	0	1	YES	I-3	0.362	0.48	0.158	NO
M-1	0.008	0.529	0.463	YES	M-1	0.565	0.283	0.151	YES
M-2	0.005	0.611	0.383	YES	M-2	0	1	0	NO
M-3	0	0.555	0.445	YES	M-3	0.128	0.756	0.116	NO
M-4	0.039	0.471	0.49	YES	M-4	0.267	0.604	0.129	YES
M-5	0.003	0.535	0.462	YES	M-5	0	1	0	NO
M-6	0.085	0.435	0.479	YES	M-6	0	1	0	NO
M-7	0.127	0.444	0.43	YES	M-7	0.406	0.396	0.198	YES
M-8	0.162	0.421	0.417	YES	M-8	0.565	0.283	0.152	YES

**Table 6:** Characteristics of overlapping SNP loci between RNA-seq datasets and the 25k SNP array. This table summarizes SNP loci shared between RNA-seq-derived datasets (ALL and FINAL) and the 25k SNP array. For each locus, chromosomal position, allele composition in RNA-seq and SNP array data, and genotype concordance category are shown. Concordance categories include exact match, reverse complement concordance, and strand swap (discordant orientation). Gene annotations are provided based on maize genome assemblies v3, v4, and v5, along with inferred gene function.

CHR	POS	RNA_REFALT	ARRAY_REFALT	Match_Type	25k Array SNP ID	Gene ID Zm_v3	Gene ID Zm_v4	Gene ID Zm_v5	Annotation
2	136193	G/A	C/T	reverse_complement	ZmSYNBREED_23550_437	*GRMZM2G046590*	*Zm00001d001765*	*Zm00001eb001180*	EXO70A1
2	214661090	C/T	A/G	reverse_complement_swap	PZE-102171277	*GRMZM2G022947*	*Zm00001d007044*	*Zm00001eb071610*	CYP727A4/DUF (domen of unknown function)
2	232838881	C/A	C/T	mismatch	PZE-102189186	*GRMZM2G105652*	*Zm00001d005110*	*Zm00001eb015110*	ZmMTP1-1
5	2795466	A/G	A/G	direct	ZmSYNBREED_45130_180	*GRMZM2G108716*	*Zm00001d012993*	*Zm00001eb212040*	IWS1/SPN1 transcription factor
9	314644	T/G	G/T	swap	ZmSYNBREED_66628_477	*GRMZM2G310569*	*Zm00001d044717*	*Zm00004b031450*	potassium outward rectifying channel
9	20238182	T/A	C/T	mismatch	PZE-109019829	*GRMZM2G028928*	*Zm00001d022088*	*Zm00001eb327040*	mads67 transcription factor

## Data Availability

The original contributions presented in this study are included in the article/Supplementary Material. Further inquiries can be directed to the corresponding author.

## Supplementary Materials

The following supporting information can be downloaded at: https://www.mdpi.com/article/10.3390/cimb48060586/s1.

## Abbreviations

The following abbreviations are used in this manuscript:

MRIZP	Maize Research Institute “Zemun Polje”, Belgrade, Serbia
SNP	Single nucleotide polymorphism
25k SNP array	25k Illumina^®^ Infinum Maize SNP Array
RNA-seq	Whole-transcriptome sequencing (RNA sequencing)
PCoA	Principal coordinate analysis
