# Supplementary material for: Genetic Relatedness and Heterotic Grouping in MRIZP Elite Maize Inbred Lines Using SNP Markers from 25k SNP Array and RNA-seq Data
Source: Curr Issues Mol Biol. 2026 Jun 2;48(6):586. doi: 10.3390/cimb48060586 (PMC13298533; doi:10.3390/cimb48060586)
Supplement: Supplementary file 1 [file cimb-48-00586-s001.zip › cimb-4191867-supplementary metarials 6.10.pdf]

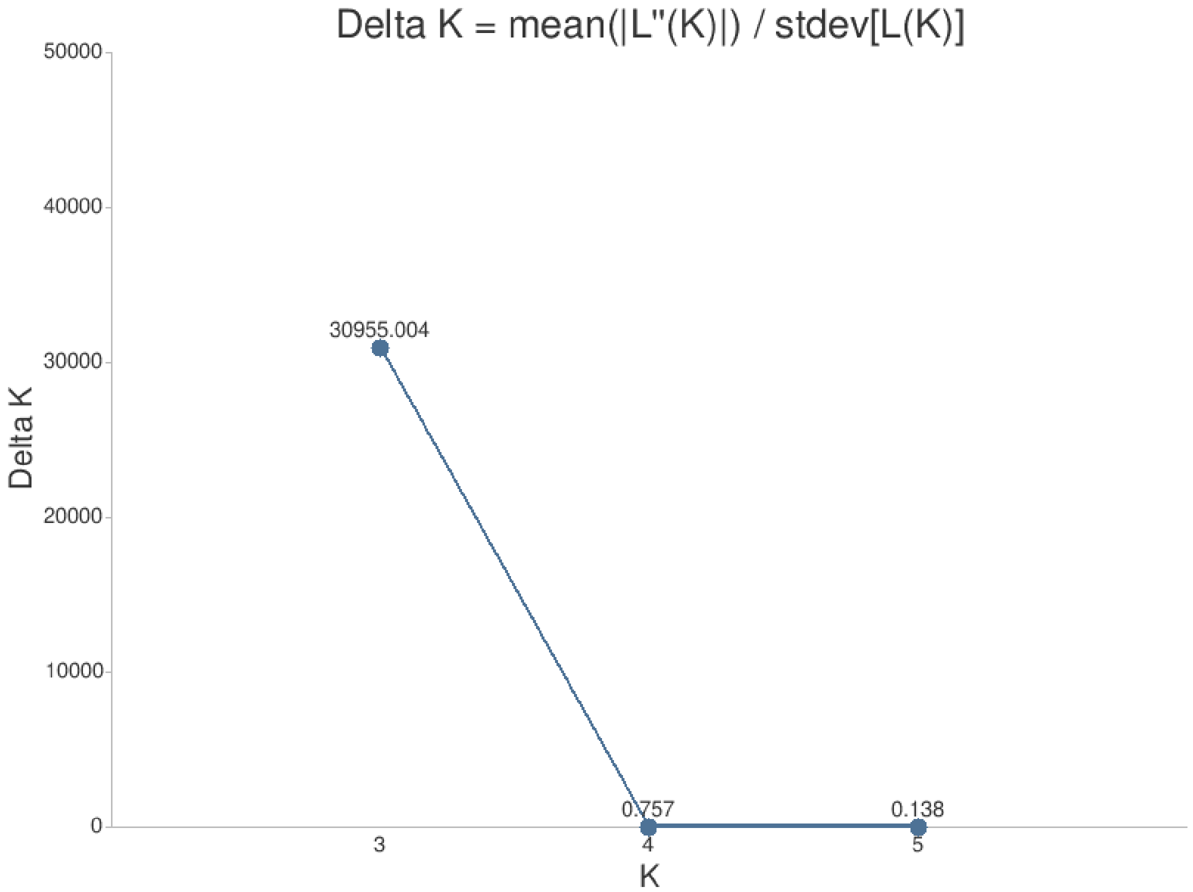

Supplementary Figure S1. Delta K (ΔK) plot for determining the optimal number of clusters (K) for 25k array-based SNPs

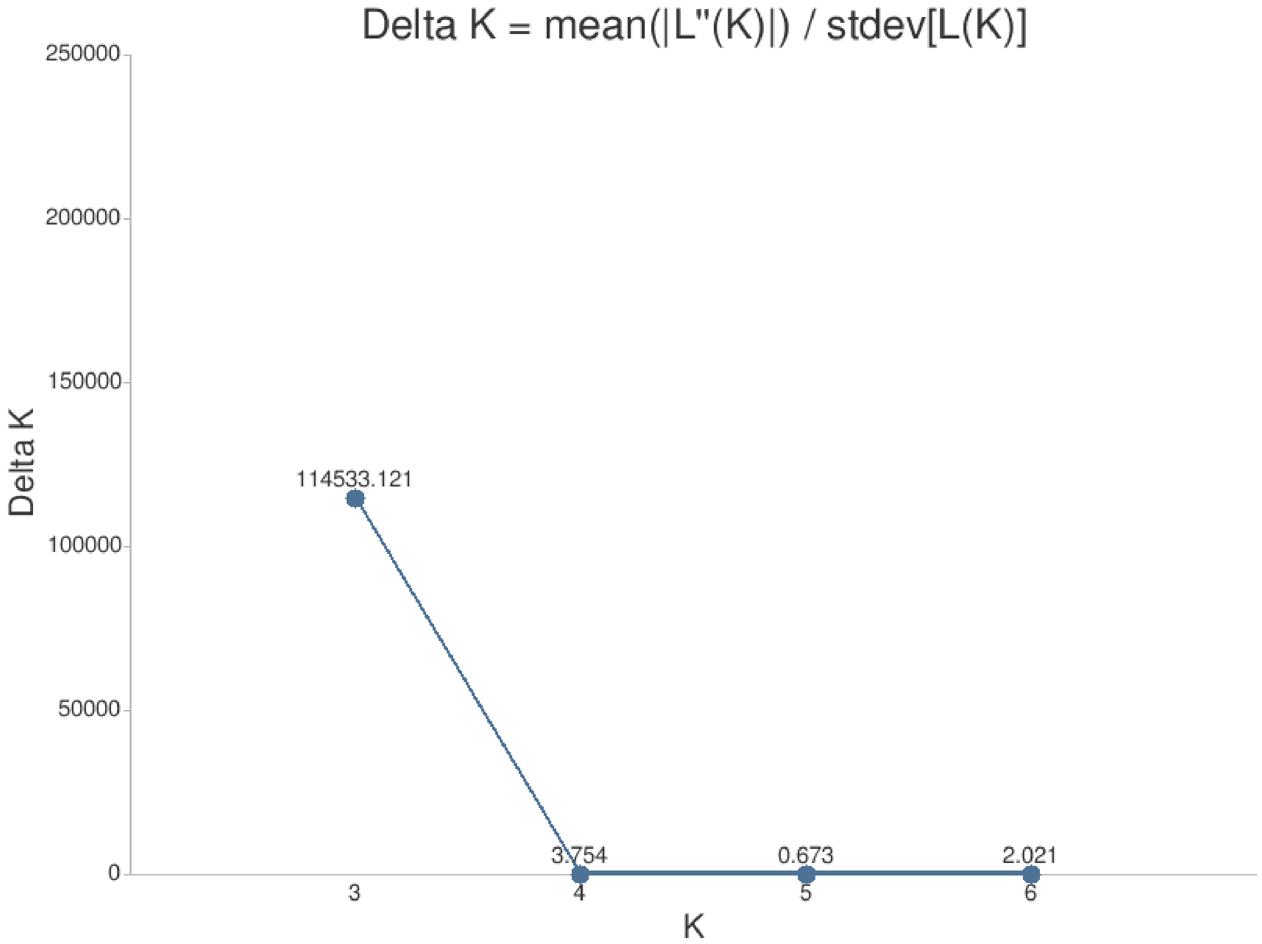

Supplementary Figure S2. Delta K (ΔK) plot for determining the optimal number of clusters (K) for RNA-seq-derived SNPs

Supplementary Table S1. LD pruning sensitivity grid across r² thresholds and window sizes, including a non-LD baseline (max_missing = 0.5).

max_missing	min_shared	mode	ld_r2	window_bp	window_snps	n_rna_postfilter	n_arr_postfilter	n_rna_used	n_arr_used	permanova_rna_R2	permanova_rna_p	permanova_arr_R2	permanova_arr_p	mantel_r	mantel_p	arr_frac_pos0	arr_frac_pos_invalid
0.5	100	noLD	NA	NA	50	22396	15885	22396	15885	0.418365982	0.0001	0.558131888	0.0001	0.621516903	0.0001	0	0
0.5	100	LD	0.1	100000	50	22396	15885	7041	8979	0.430093592	0.0001	0.559628006	0.0001	0.639198702	0.0001	0	0
0.5	100	LD	0.1	500000	50	22396	15885	3874	4017	0.431108555	0.0001	0.559543464	0.0001	0.638304336	0.0001	0	0
0.5	100	LD	0.2	100000	50	22396	15885	7976	10189	0.430786282	0.0001	0.549668592	0.0001	0.639840986	0.0001	0	0
0.5	100	LD	0.2	500000	50	22396	15885	4794	5546	0.438084887	0.0001	0.534912036	0.0001	0.64428548	0.0001	0	0
0.5	100	LD	0.5	100000	50	22396	15885	9949	12129	0.428052212	0.0001	0.549225098	0.0001	0.642330562	0.0001	0	0
0.5	100	LD	0.5	500000	50	22396	15885	7155	8954	0.431019126	0.0001	0.526948503	0.0001	0.642349711	0.0001	0	0

Supplementary Table S2. Pairwise distance matrix of 30 evaluated inbred lines calculated from 25k array-based SNPs

Inbred line	Mo17	L-1	L-2	L-3	L-4	L-5	L-6	L-7	L-8	L-9	B73	B-1	B-2	B-3	B-4	B-5	B-6	B-7	B-8	I-1	I-2	I-3	M-1	M-2	M-3	M-4	M-5	M-6	M-7	M-8
**Mo17**	/	0.218	0.215	0.138	0.162	0.272	0.191	0.262	0.137	0.184	0.499	0.485	0.470	0.477	0.491	0.489	0.476	0.480	0.486	0.469	0.460	0.453	0.478	0.473	0.471	0.464	0.480	0.446	0.455	0.437
**L-1**	0.218	/	0.028	0.209	0.219	0.347	0.290	0.202	0.238	0.163	0.498	0.484	0.453	0.469	0.485	0.482	0.461	0.474	0.479	0.485	0.476	0.471	0.480	0.480	0.481	0.474	0.480	0.439	0.441	0.434
**L-2**	0.215	0.028	/	0.202	0.216	0.349	0.289	0.213	0.231	0.147	0.497	0.481	0.456	0.472	0.487	0.485	0.463	0.477	0.481	0.486	0.476	0.471	0.478	0.480	0.480	0.473	0.479	0.440	0.444	0.441
**L-3**	0.138	0.209	0.202	/	0.117	0.246	0.155	0.277	0.179	0.136	0.501	0.486	0.465	0.472	0.484	0.487	0.465	0.473	0.480	0.472	0.463	0.458	0.479	0.473	0.474	0.467	0.473	0.448	0.448	0.444
**L-4**	0.162	0.219	0.216	0.117	/	0.267	0.157	0.303	0.199	0.069	0.498	0.485	0.468	0.471	0.483	0.486	0.472	0.475	0.478	0.469	0.466	0.456	0.482	0.475	0.481	0.470	0.479	0.445	0.448	0.440
**L-5**	0.272	0.347	0.349	0.246	0.267	/	0.196	0.383	0.336	0.301	0.492	0.458	0.439	0.429	0.468	0.465	0.451	0.459	0.464	0.460	0.462	0.457	0.475	0.463	0.473	0.462	0.479	0.430	0.427	0.433
**L-6**	0.191	0.290	0.289	0.155	0.157	0.196	/	0.343	0.276	0.200	0.500	0.488	0.471	0.470	0.477	0.493	0.464	0.470	0.476	0.466	0.461	0.462	0.483	0.475	0.481	0.470	0.479	0.450	0.449	0.445
**L-7**	0.262	0.202	0.213	0.277	0.303	0.383	0.343	/	0.228	0.266	0.479	0.468	0.439	0.460	0.474	0.467	0.453	0.461	0.464	0.474	0.463	0.458	0.465	0.466	0.465	0.463	0.474	0.433	0.440	0.431
**L-8**	0.137	0.238	0.231	0.179	0.199	0.336	0.276	0.228	/	0.198	0.492	0.470	0.457	0.473	0.488	0.483	0.470	0.474	0.478	0.463	0.454	0.449	0.464	0.457	0.461	0.455	0.465	0.454	0.452	0.445
**L-9**	0.184	0.163	0.147	0.136	0.069	0.301	0.200	0.266	0.198	/	0.498	0.482	0.470	0.476	0.482	0.491	0.473	0.474	0.482	0.474	0.468	0.458	0.481	0.477	0.480	0.470	0.477	0.451	0.451	0.443
**B73**	0.499	0.498	0.497	0.501	0.498	0.492	0.500	0.479	0.492	0.498	/	0.260	0.306	0.394	0.393	0.341	0.327	0.362	0.251	0.468	0.461	0.461	0.387	0.379	0.357	0.343	0.387	0.424	0.451	0.456
**B-1**	0.485	0.484	0.481	0.486	0.485	0.458	0.488	0.468	0.470	0.482	0.260	/	0.363	0.406	0.381	0.385	0.386	0.375	0.352	0.451	0.449	0.447	0.405	0.394	0.391	0.400	0.407	0.423	0.454	0.432
**B-2**	0.470	0.453	0.456	0.465	0.468	0.439	0.471	0.439	0.457	0.470	0.306	0.363	/	0.379	0.370	0.143	0.149	0.340	0.334	0.475	0.475	0.469	0.429	0.397	0.421	0.432	0.432	0.426	0.438	0.411
**B-3**	0.477	0.469	0.472	0.472	0.471	0.429	0.470	0.460	0.473	0.476	0.394	0.406	0.379	/	0.290	0.394	0.345	0.271	0.302	0.424	0.423	0.421	0.382	0.379	0.398	0.406	0.419	0.392	0.394	0.431
**B-4**	0.491	0.485	0.487	0.484	0.483	0.468	0.477	0.474	0.488	0.482	0.393	0.381	0.370	0.290	/	0.390	0.251	0.102	0.229	0.457	0.459	0.466	0.416	0.380	0.412	0.443	0.435	0.405	0.408	0.419
**B-5**	0.489	0.482	0.485	0.487	0.486	0.465	0.493	0.467	0.483	0.491	0.341	0.385	0.143	0.394	0.390	/	0.249	0.389	0.378	0.395	0.400	0.385	0.390	0.409	0.368	0.400	0.417	0.393	0.428	0.396
**B-6**	0.476	0.461	0.463	0.465	0.472	0.451	0.464	0.453	0.470	0.473	0.327	0.386	0.149	0.345	0.251	0.249	/	0.193	0.223	0.468	0.466	0.473	0.429	0.389	0.410	0.434	0.418	0.416	0.420	0.420
**B-7**	0.480	0.474	0.477	0.473	0.475	0.459	0.470	0.461	0.474	0.474	0.362	0.375	0.340	0.271	0.102	0.389	0.193	/	0.145	0.464	0.469	0.474	0.416	0.375	0.407	0.442	0.424	0.377	0.388	0.399
**B-8**	0.486	0.479	0.481	0.480	0.478	0.464	0.476	0.464	0.478	0.482	0.251	0.352	0.334	0.302	0.229	0.378	0.223	0.145	/	0.464	0.464	0.468	0.425	0.370	0.399	0.406	0.405	0.382	0.395	0.405
**I-1**	0.469	0.485	0.486	0.472	0.469	0.460	0.466	0.474	0.463	0.474	0.468	0.451	0.475	0.424	0.457	0.395	0.468	0.464	0.464	/	0.070	0.104	0.351	0.362	0.338	0.337	0.339	0.308	0.341	0.331
**I-2**	0.460	0.476	0.476	0.463	0.466	0.462	0.461	0.463	0.454	0.468	0.461	0.449	0.475	0.423	0.459	0.400	0.466	0.469	0.464	0.070	/	0.078	0.348	0.356	0.354	0.328	0.353	0.324	0.356	0.345
**I-3**	0.453	0.471	0.471	0.458	0.456	0.457	0.462	0.458	0.449	0.458	0.461	0.447	0.469	0.421	0.466	0.385	0.473	0.474	0.468	0.104	0.078	/	0.323	0.339	0.328	0.300	0.331	0.301	0.323	0.341
**M-1**	0.478	0.480	0.478	0.479	0.482	0.475	0.483	0.465	0.464	0.481	0.387	0.405	0.429	0.382	0.416	0.390	0.429	0.416	0.425	0.351	0.348	0.323	/	0.304	0.236	0.238	0.284	0.371	0.415	0.414
**M-2**	0.473	0.480	0.480	0.473	0.475	0.463	0.475	0.466	0.457	0.477	0.379	0.394	0.397	0.379	0.380	0.409	0.389	0.375	0.370	0.362	0.356	0.339	0.304	/	0.262	0.338	0.296	0.381	0.398	0.409
**M-3**	0.471	0.481	0.480	0.474	0.481	0.473	0.481	0.465	0.461	0.480	0.357	0.391	0.421	0.398	0.412	0.368	0.410	0.407	0.399	0.338	0.354	0.328	0.236	0.262	/	0.279	0.225	0.354	0.394	0.410
**M-4**	0.464	0.474	0.473	0.467	0.470	0.462	0.470	0.463	0.455	0.470	0.343	0.400	0.432	0.406	0.443	0.400	0.434	0.442	0.406	0.337	0.328	0.300	0.238	0.338	0.279	/	0.342	0.391	0.395	0.405
**M-5**	0.480	0.480	0.479	0.473	0.479	0.479	0.479	0.474	0.465	0.477	0.387	0.407	0.432	0.419	0.435	0.417	0.418	0.424	0.405	0.339	0.353	0.331	0.284	0.296	0.225	0.342	/	0.349	0.345	0.388
**M-6**	0.446	0.439	0.440	0.448	0.445	0.430	0.450	0.433	0.454	0.451	0.424	0.423	0.426	0.392	0.405	0.393	0.416	0.377	0.382	0.308	0.324	0.301	0.371	0.381	0.354	0.391	0.349	/	0.233	0.342

**M-7**	0.455	0.441	0.444	0.448	0.448	0.427	0.449	0.440	0.452	0.451	0.451	0.454	0.438	0.394	0.408	0.428	0.420	0.388	0.395	0.341	0.356	0.323	0.415	0.398	0.394	0.395	0.345	0.233	/	0.370
**M-8**	0.437	0.434	0.441	0.444	0.440	0.433	0.445	0.431	0.445	0.443	0.456	0.432	0.411	0.431	0.419	0.396	0.420	0.399	0.405	0.331	0.345	0.341	0.414	0.409	0.410	0.405	0.388	0.342	0.370	/

Inbred line	**Mo17**	**L-1**	**L-2**	**L-3**	**L-4**	**L-5**	**L-6**	**L-7**	**L-8**	**L-9**	**B73**	**B-1**	**B-2**	**B-3**	**B-4**	**B-5**	**B-6**	**B-7**	**B-8**	**I-1**	**I-2**	**I-3**	**M-1**	**M-2**	**M-3**	**M-4**	**M-5**	**M-6**	**M-7**	**M-8**
**Mo17**	/	0.218	0.215	0.138	0.162	0.272	0.191	0.262	0.137	0.184	0.499	0.485	0.470	0.477	0.491	0.489	0.476	0.480	0.486	0.469	0.460	0.453	0.478	0.473	0.471	0.464	0.480	0.446	0.455	0.437
**L-1**	0.218	/	0.028	0.209	0.219	0.347	0.290	0.202	0.238	0.163	0.498	0.484	0.453	0.469	0.485	0.482	0.461	0.474	0.479	0.485	0.476	0.471	0.480	0.480	0.481	0.474	0.480	0.439	0.441	0.434
**L-2**	0.215	0.028	/	0.202	0.216	0.349	0.289	0.213	0.231	0.147	0.497	0.481	0.456	0.472	0.487	0.485	0.463	0.477	0.481	0.486	0.476	0.471	0.478	0.480	0.480	0.473	0.479	0.440	0.444	0.441
**L-3**	0.138	0.209	0.202	/	0.117	0.246	0.155	0.277	0.179	0.136	0.501	0.486	0.465	0.472	0.484	0.487	0.465	0.473	0.480	0.472	0.463	0.458	0.479	0.473	0.474	0.467	0.473	0.448	0.448	0.444
**L-4**	0.162	0.219	0.216	0.117	/	0.267	0.157	0.303	0.199	0.069	0.498	0.485	0.468	0.471	0.483	0.486	0.472	0.475	0.478	0.469	0.466	0.456	0.482	0.475	0.481	0.470	0.479	0.445	0.448	0.440
**L-5**	0.272	0.347	0.349	0.246	0.267	/	0.196	0.383	0.336	0.301	0.492	0.458	0.439	0.429	0.468	0.465	0.451	0.459	0.464	0.460	0.462	0.457	0.475	0.463	0.473	0.462	0.479	0.430	0.427	0.433
**L-6**	0.191	0.290	0.289	0.155	0.157	0.196	/	0.343	0.276	0.200	0.500	0.488	0.471	0.470	0.477	0.493	0.464	0.470	0.476	0.466	0.461	0.462	0.483	0.475	0.481	0.470	0.479	0.450	0.449	0.445
**L-7**	0.262	0.202	0.213	0.277	0.303	0.383	0.343	/	0.228	0.266	0.479	0.468	0.439	0.460	0.474	0.467	0.453	0.461	0.464	0.474	0.463	0.458	0.465	0.466	0.465	0.463	0.474	0.433	0.440	0.431
**L-8**	0.137	0.238	0.231	0.179	0.199	0.336	0.276	0.228	/	0.198	0.492	0.470	0.457	0.473	0.488	0.483	0.470	0.474	0.478	0.463	0.454	0.449	0.464	0.457	0.461	0.455	0.465	0.454	0.452	0.445
**L-9**	0.184	0.163	0.147	0.136	0.069	0.301	0.200	0.266	0.198	/	0.498	0.482	0.470	0.476	0.482	0.491	0.473	0.474	0.482	0.474	0.468	0.458	0.481	0.477	0.480	0.470	0.477	0.451	0.451	0.443
**B73**	0.499	0.498	0.497	0.501	0.498	0.492	0.500	0.479	0.492	0.498	/	0.260	0.306	0.394	0.393	0.341	0.327	0.362	0.251	0.468	0.461	0.461	0.387	0.379	0.357	0.343	0.387	0.424	0.451	0.456
**B-1**	0.485	0.484	0.481	0.486	0.485	0.458	0.488	0.468	0.470	0.482	0.260	/	0.363	0.406	0.381	0.385	0.386	0.375	0.352	0.451	0.449	0.447	0.405	0.394	0.391	0.400	0.407	0.423	0.454	0.432
**B-2**	0.470	0.453	0.456	0.465	0.468	0.439	0.471	0.439	0.457	0.470	0.306	0.363	/	0.379	0.370	0.143	0.149	0.340	0.334	0.475	0.475	0.469	0.429	0.397	0.421	0.432	0.432	0.426	0.438	0.411
**B-3**	0.477	0.469	0.472	0.472	0.471	0.429	0.470	0.460	0.473	0.476	0.394	0.406	0.379	/	0.290	0.394	0.345	0.271	0.302	0.424	0.423	0.421	0.382	0.379	0.398	0.406	0.419	0.392	0.394	0.431
**B-4**	0.491	0.485	0.487	0.484	0.483	0.468	0.477	0.474	0.488	0.482	0.393	0.381	0.370	0.290	/	0.390	0.251	0.102	0.229	0.457	0.459	0.466	0.416	0.380	0.412	0.443	0.435	0.405	0.408	0.419
**B-5**	0.489	0.482	0.485	0.487	0.486	0.465	0.493	0.467	0.483	0.491	0.341	0.385	0.143	0.394	0.390	/	0.249	0.389	0.378	0.395	0.400	0.385	0.390	0.409	0.368	0.400	0.417	0.393	0.428	0.396
**B-6**	0.476	0.461	0.463	0.465	0.472	0.451	0.464	0.453	0.470	0.473	0.327	0.386	0.149	0.345	0.251	0.249	/	0.193	0.223	0.468	0.466	0.473	0.429	0.389	0.410	0.434	0.418	0.416	0.420	0.420
**B-7**	0.480	0.474	0.477	0.473	0.475	0.459	0.470	0.461	0.474	0.474	0.362	0.375	0.340	0.271	0.102	0.389	0.193	/	0.145	0.464	0.469	0.474	0.416	0.375	0.407	0.442	0.424	0.377	0.388	0.399
**B-8**	0.486	0.479	0.481	0.480	0.478	0.464	0.476	0.464	0.478	0.482	0.251	0.352	0.334	0.302	0.229	0.378	0.223	0.145	/	0.464	0.464	0.468	0.425	0.370	0.399	0.406	0.405	0.382	0.395	0.405
**I-1**	0.469	0.485	0.486	0.472	0.469	0.460	0.466	0.474	0.463	0.474	0.468	0.451	0.475	0.424	0.457	0.395	0.468	0.464	0.464	/	0.070	0.104	0.351	0.362	0.338	0.337	0.339	0.308	0.341	0.331
**I-2**	0.460	0.476	0.476	0.463	0.466	0.462	0.461	0.463	0.454	0.468	0.461	0.449	0.475	0.423	0.459	0.400	0.466	0.469	0.464	0.070	/	0.078	0.348	0.356	0.354	0.328	0.353	0.324	0.356	0.345
**I-3**	0.453	0.471	0.471	0.458	0.456	0.457	0.462	0.458	0.449	0.458	0.461	0.447	0.469	0.421	0.466	0.385	0.473	0.474	0.468	0.104	0.078	/	0.323	0.339	0.328	0.300	0.331	0.301	0.323	0.341
**M-1**	0.478	0.480	0.478	0.479	0.482	0.475	0.483	0.465	0.464	0.481	0.387	0.405	0.429	0.382	0.416	0.390	0.429	0.416	0.425	0.351	0.348	0.323	/	0.304	0.236	0.238	0.284	0.371	0.415	0.414
**M-2**	0.473	0.480	0.480	0.473	0.475	0.463	0.475	0.466	0.457	0.477	0.379	0.394	0.397	0.379	0.380	0.409	0.389	0.375	0.370	0.362	0.356	0.339	0.304	/	0.262	0.338	0.296	0.381	0.398	0.409
**M-3**	0.471	0.481	0.480	0.474	0.481	0.473	0.481	0.465	0.461	0.480	0.357	0.391	0.421	0.398	0.412	0.368	0.410	0.407	0.399	0.338	0.354	0.328	0.236	0.262	/	0.279	0.225	0.354	0.394	0.410
**M-4**	0.464	0.474	0.473	0.467	0.470	0.462	0.470	0.463	0.455	0.470	0.343	0.400	0.432	0.406	0.443	0.400	0.434	0.442	0.406	0.337	0.328	0.300	0.238	0.338	0.279	/	0.342	0.391	0.395	0.405
**M-5**	0.480	0.480	0.479	0.473	0.479	0.479	0.479	0.474	0.465	0.477	0.387	0.407	0.432	0.419	0.435	0.417	0.418	0.424	0.405	0.339	0.353	0.331	0.284	0.296	0.225	0.342	/	0.349	0.345	0.388
**M-6**	0.446	0.439	0.440	0.448	0.445	0.430	0.450	0.433	0.454	0.451	0.424	0.423	0.426	0.392	0.405	0.393	0.416	0.377	0.382	0.308	0.324	0.301	0.371	0.381	0.354	0.391	0.349	/	0.233	0.342
**M-7**	0.455	0.441	0.444	0.448	0.448	0.427	0.449	0.440	0.452	0.451	0.451	0.454	0.438	0.394	0.408	0.428	0.420	0.388	0.395	0.341	0.356	0.323	0.415	0.398	0.394	0.395	0.345	0.233	/	0.370
**M-8**	0.437	0.434	0.441	0.444	0.440	0.433	0.445	0.431	0.445	0.443	0.456	0.432	0.411	0.431	0.419	0.396	0.420	0.399	0.405	0.331	0.345	0.341	0.414	0.409	0.410	0.405	0.388	0.342	0.370	/

Supplementary Table S3. Pairwise distance matrix of 30 evaluated inbred lines calculated from RNA-seq-derived SNPs

Inbred line	Mo17	L-1	L-2	L-3	L-4	L-5	L-6	L-7	L-8	L-9	B73	B-1	B-2	B-3	B-4	B-5	B-6	B-7	B-8	I-1	I-2	I-3	M-1	M-2	M-3	M-4	M-5	M-6	M-7	M-8
**Mo17**	/	0.183	0.162	0.099	0.104	0.190	0.137	0.193	0.084	0.095	0.435	0.426	0.402	0.423	0.363	0.451	0.423	0.343	0.400	0.451	0.399	0.335	0.365	0.399	0.399	0.388	0.426	0.435	0.300	0.365
**L-1**	0.183	/	0.069	0.151	0.165	0.256	0.229	0.153	0.143	0.102	0.445	0.452	0.401	0.419	0.367	0.375	0.419	0.366	0.420	0.375	0.424	0.351	0.382	0.424	0.415	0.418	0.452	0.445	0.307	0.382
**L-2**	0.162	0.069	/	0.155	0.158	0.245	0.228	0.170	0.120	0.097	0.340	0.355	0.329	0.427	0.354	0.438	0.427	0.312	0.376	0.438	0.236	0.312	0.359	0.236	0.339	0.358	0.355	0.340	0.262	0.359
**L-3**	0.099	0.151	0.155	/	0.070	0.164	0.103	0.190	0.083	0.068	0.409	0.403	0.374	0.428	0.357	0.440	0.428	0.329	0.403	0.440	0.383	0.333	0.358	0.383	0.393	0.388	0.403	0.409	0.282	0.358
**L-4**	0.104	0.165	0.158	0.070	/	0.178	0.103	0.198	0.104	0.035	0.423	0.410	0.401	0.422	0.353	0.435	0.422	0.319	0.388	0.435	0.394	0.337	0.365	0.394	0.396	0.397	0.410	0.423	0.289	0.365
**L-5**	0.190	0.256	0.245	0.164	0.178	/	0.138	0.284	0.179	0.154	0.400	0.404	0.355	0.411	0.347	0.418	0.411	0.321	0.389	0.418	0.381	0.323	0.354	0.381	0.391	0.370	0.404	0.400	0.275	0.354
**L-6**	0.137	0.229	0.228	0.103	0.103	0.138	/	0.263	0.169	0.121	0.439	0.431	0.422	0.440	0.395	0.466	0.440	0.363	0.431	0.466	0.421	0.354	0.386	0.421	0.423	0.410	0.431	0.439	0.325	0.386
**L-7**	0.193	0.153	0.170	0.190	0.198	0.284	0.263	/	0.126	0.143	0.420	0.425	0.394	0.432	0.379	0.439	0.432	0.352	0.408	0.439	0.406	0.340	0.381	0.406	0.412	0.401	0.425	0.420	0.305	0.381
**L-8**	0.084	0.143	0.120	0.083	0.104	0.179	0.169	0.126	/	0.066	0.345	0.342	0.328	0.369	0.277	0.385	0.369	0.231	0.307	0.385	0.320	0.271	0.336	0.320	0.316	0.320	0.342	0.345	0.216	0.336
**L-9**	0.095	0.102	0.097	0.068	0.035	0.154	0.121	0.143	0.066	/	0.372	0.362	0.340	0.362	0.279	0.389	0.362	0.243	0.311	0.389	0.328	0.276	0.333	0.328	0.338	0.336	0.362	0.372	0.221	0.333
**B73**	0.435	0.445	0.340	0.409	0.423	0.400	0.439	0.420	0.345	0.372	/	0.147	0.290	0.445	0.379	0.410	0.445	0.310	0.374	0.410	0.218	0.287	0.375	0.218	0.209	0.301	0.147	0.000	0.279	0.375
**B-1**	0.426	0.452	0.355	0.403	0.410	0.404	0.431	0.425	0.342	0.362	0.147	/	0.238	0.467	0.400	0.433	0.467	0.347	0.400	0.433	0.259	0.305	0.417	0.259	0.251	0.313	0.000	0.147	0.301	0.417
**B-2**	0.402	0.401	0.329	0.374	0.401	0.355	0.422	0.394	0.328	0.340	0.290	0.238	/	0.450	0.385	0.393	0.450	0.348	0.402	0.393	0.271	0.269	0.401	0.271	0.272	0.276	0.238	0.290	0.282	0.401
**B-3**	0.423	0.419	0.427	0.428	0.422	0.411	0.440	0.432	0.369	0.362	0.445	0.467	0.450	/	0.017	0.282	0.000	0.157	0.224	0.282	0.428	0.413	0.453	0.428	0.434	0.463	0.467	0.445	0.349	0.453
**B-4**	0.363	0.367	0.354	0.357	0.353	0.347	0.395	0.379	0.277	0.279	0.379	0.400	0.385	0.017	/	0.234	0.017	0.113	0.177	0.234	0.358	0.339	0.393	0.358	0.370	0.395	0.400	0.379	0.272	0.393
**B-5**	0.451	0.375	0.438	0.440	0.435	0.418	0.466	0.439	0.385	0.389	0.410	0.433	0.393	0.282	0.234	/	0.282	0.340	0.378	0.000	0.421	0.330	0.404	0.421	0.371	0.404	0.433	0.410	0.347	0.404
**B-6**	0.423	0.419	0.427	0.428	0.422	0.411	0.440	0.432	0.369	0.362	0.445	0.467	0.450	0.000	0.017	0.282	/	0.157	0.224	0.282	0.428	0.413	0.453	0.428	0.434	0.463	0.467	0.445	0.349	0.453
**B-7**	0.343	0.366	0.312	0.329	0.319	0.321	0.363	0.352	0.231	0.243	0.310	0.347	0.348	0.157	0.113	0.340	0.157	/	0.103	0.340	0.299	0.270	0.328	0.299	0.328	0.361	0.347	0.310	0.232	0.328
**B-8**	0.400	0.420	0.376	0.403	0.388	0.389	0.431	0.408	0.307	0.311	0.374	0.400	0.402	0.224	0.177	0.378	0.224	0.103	/	0.378	0.356	0.359	0.400	0.356	0.384	0.396	0.400	0.374	0.296	0.400
**I-1**	0.451	0.375	0.438	0.440	0.435	0.418	0.466	0.439	0.385	0.389	0.410	0.433	0.393	0.282	0.234	0.000	0.282	0.340	0.378	/	0.421	0.330	0.404	0.421	0.371	0.404	0.433	0.410	0.347	0.404
**I-2**	0.399	0.424	0.236	0.383	0.394	0.381	0.421	0.406	0.320	0.328	0.218	0.259	0.271	0.428	0.358	0.421	0.428	0.299	0.356	0.421	/	0.299	0.390	0.000	0.257	0.296	0.259	0.218	0.290	0.390
**I-3**	0.335	0.351	0.312	0.333	0.337	0.323	0.354	0.340	0.271	0.276	0.287	0.305	0.269	0.413	0.339	0.330	0.413	0.270	0.359	0.330	0.299	/	0.263	0.299	0.262	0.261	0.305	0.287	0.152	0.263
**M-1**	0.365	0.382	0.359	0.358	0.365	0.354	0.386	0.381	0.336	0.333	0.375	0.417	0.401	0.453	0.393	0.404	0.453	0.328	0.400	0.404	0.390	0.263	/	0.390	0.373	0.370	0.417	0.375	0.295	0.000
**M-2**	0.399	0.424	0.236	0.383	0.394	0.381	0.421	0.406	0.320	0.328	0.218	0.259	0.271	0.428	0.358	0.421	0.428	0.299	0.356	0.421	0.000	0.299	0.390	/	0.257	0.296	0.259	0.218	0.290	0.390
**M-3**	0.399	0.415	0.339	0.393	0.396	0.391	0.423	0.412	0.316	0.338	0.209	0.251	0.272	0.434	0.370	0.371	0.434	0.328	0.384	0.371	0.257	0.262	0.373	0.257	/	0.268	0.251	0.209	0.299	0.373
**M-4**	0.388	0.418	0.358	0.388	0.397	0.370	0.410	0.401	0.320	0.336	0.301	0.313	0.276	0.463	0.395	0.404	0.463	0.361	0.396	0.404	0.296	0.261	0.370	0.296	0.268	/	0.313	0.301	0.295	0.370
**M-5**	0.426	0.452	0.355	0.403	0.410	0.404	0.431	0.425	0.342	0.362	0.147	0.000	0.238	0.467	0.400	0.433	0.467	0.347	0.400	0.433	0.259	0.305	0.417	0.259	0.251	0.313	/	0.147	0.301	0.417

**M-6**	0.435	0.445	0.340	0.409	0.423	0.400	0.439	0.420	0.345	0.372	0.000	0.147	0.290	0.445	0.379	0.410	0.445	0.310	0.374	0.410	0.218	0.287	0.375	0.218	0.209	0.301	0.147	/	0.279	0.375
**M-7**	0.300	0.307	0.262	0.282	0.289	0.275	0.325	0.305	0.216	0.221	0.279	0.301	0.282	0.349	0.272	0.347	0.349	0.232	0.296	0.347	0.290	0.152	0.295	0.290	0.299	0.295	0.301	0.279	/	0.295
**M-8**	0.365	0.382	0.359	0.358	0.365	0.354	0.386	0.381	0.336	0.333	0.375	0.417	0.401	0.453	0.393	0.404	0.453	0.328	0.400	0.404	0.390	0.263	0.000	0.390	0.373	0.370	0.417	0.375	0.295	/

Inbred line	Mo17	L-1	L-2	L-3	L-4	L-5	L-6	L-7	L-8	L-9	B73	B-1	B-2	B-3	B-4	B-5	B-6	B-7	B-8	I-1	I-2	I-3	M-1	M-2	M-3	M-4	M-5	M-6	M-7	M-8
Mo17	/	0.183	0.162	0.099	0.104	0.190	0.137	0.193	0.084	0.095	0.435	0.426	0.402	0.423	0.363	0.451	0.423	0.343	0.400	0.451	0.399	0.335	0.365	0.399	0.399	0.388	0.426	0.435	0.300	0.365
L-1	0.183	/	0.069	0.151	0.165	0.256	0.229	0.153	0.143	0.102	0.445	0.452	0.401	0.419	0.367	0.375	0.419	0.366	0.420	0.375	0.424	0.351	0.382	0.424	0.415	0.418	0.452	0.445	0.307	0.382
L-2	0.162	0.069	/	0.155	0.158	0.245	0.228	0.170	0.120	0.097	0.340	0.355	0.329	0.427	0.354	0.438	0.427	0.312	0.376	0.438	0.236	0.312	0.359	0.236	0.339	0.358	0.355	0.340	0.262	0.359
L-3	0.099	0.151	0.155	/	0.070	0.164	0.103	0.190	0.083	0.068	0.409	0.403	0.374	0.428	0.357	0.440	0.428	0.329	0.403	0.440	0.383	0.333	0.358	0.383	0.393	0.388	0.403	0.409	0.282	0.358
L-4	0.104	0.165	0.158	0.070	/	0.178	0.103	0.198	0.104	0.035	0.423	0.410	0.401	0.422	0.353	0.435	0.422	0.319	0.388	0.435	0.394	0.337	0.365	0.394	0.396	0.397	0.410	0.423	0.289	0.365
L-5	0.190	0.256	0.245	0.164	0.178	/	0.138	0.284	0.179	0.154	0.400	0.404	0.355	0.411	0.347	0.418	0.411	0.321	0.389	0.418	0.381	0.323	0.354	0.381	0.391	0.370	0.404	0.400	0.275	0.354
L-6	0.137	0.229	0.228	0.103	0.103	0.138	/	0.263	0.169	0.121	0.439	0.431	0.422	0.440	0.395	0.466	0.440	0.363	0.431	0.466	0.421	0.354	0.386	0.421	0.423	0.410	0.431	0.439	0.325	0.386
L-7	0.193	0.153	0.170	0.190	0.198	0.284	0.263	/	0.126	0.143	0.420	0.425	0.394	0.432	0.379	0.439	0.432	0.352	0.408	0.439	0.406	0.340	0.381	0.406	0.412	0.401	0.425	0.420	0.305	0.381
L-8	0.084	0.143	0.120	0.083	0.104	0.179	0.169	0.126	/	0.066	0.345	0.342	0.328	0.369	0.277	0.385	0.369	0.231	0.307	0.385	0.320	0.271	0.336	0.320	0.316	0.320	0.342	0.345	0.216	0.336
L-9	0.095	0.102	0.097	0.068	0.035	0.154	0.121	0.143	0.066	/	0.372	0.362	0.340	0.362	0.279	0.389	0.362	0.243	0.311	0.389	0.328	0.276	0.333	0.328	0.338	0.336	0.362	0.372	0.221	0.333
B73	0.435	0.445	0.340	0.409	0.423	0.400	0.439	0.420	0.345	0.372	/	0.147	0.290	0.445	0.379	0.410	0.445	0.310	0.374	0.410	0.218	0.287	0.375	0.218	0.209	0.301	0.147	0.000	0.279	0.375
B-1	0.426	0.452	0.355	0.403	0.410	0.404	0.431	0.425	0.342	0.362	0.147	/	0.238	0.467	0.400	0.433	0.467	0.347	0.400	0.433	0.259	0.305	0.417	0.259	0.251	0.313	0.000	0.147	0.301	0.417
B-2	0.402	0.401	0.329	0.374	0.401	0.355	0.422	0.394	0.328	0.340	0.290	0.238	/	0.450	0.385	0.393	0.450	0.348	0.402	0.393	0.271	0.269	0.401	0.271	0.272	0.276	0.238	0.290	0.282	0.401
B-3	0.423	0.419	0.427	0.428	0.422	0.411	0.440	0.432	0.369	0.362	0.445	0.467	0.450	/	0.017	0.282	0.000	0.157	0.224	0.282	0.428	0.413	0.453	0.428	0.434	0.463	0.467	0.445	0.349	0.453
B-4	0.363	0.367	0.354	0.357	0.353	0.347	0.395	0.379	0.277	0.279	0.379	0.400	0.385	0.017	/	0.234	0.017	0.113	0.177	0.234	0.358	0.339	0.393	0.358	0.370	0.395	0.400	0.379	0.272	0.393
B-5	0.451	0.375	0.438	0.440	0.435	0.418	0.466	0.439	0.385	0.389	0.410	0.433	0.393	0.282	0.234	/	0.282	0.340	0.378	0.000	0.421	0.330	0.404	0.421	0.371	0.404	0.433	0.410	0.347	0.404
B-6	0.423	0.419	0.427	0.428	0.422	0.411	0.440	0.432	0.369	0.362	0.445	0.467	0.450	0.000	0.017	0.282	/	0.157	0.224	0.282	0.428	0.413	0.453	0.428	0.434	0.463	0.467	0.445	0.349	0.453
B-7	0.343	0.366	0.312	0.329	0.319	0.321	0.363	0.352	0.231	0.243	0.310	0.347	0.348	0.157	0.113	0.340	0.157	/	0.103	0.340	0.299	0.270	0.328	0.299	0.328	0.361	0.347	0.310	0.232	0.328
B-8	0.400	0.420	0.376	0.403	0.388	0.389	0.431	0.408	0.307	0.311	0.374	0.400	0.402	0.224	0.177	0.378	0.224	0.103	/	0.378	0.356	0.359	0.400	0.356	0.384	0.396	0.400	0.374	0.296	0.400
I-1	0.451	0.375	0.438	0.440	0.435	0.418	0.466	0.439	0.385	0.389	0.410	0.433	0.393	0.282	0.234	0.000	0.282	0.340	0.378	/	0.421	0.330	0.404	0.421	0.371	0.404	0.433	0.410	0.347	0.404
I-2	0.399	0.424	0.236	0.383	0.394	0.381	0.421	0.406	0.320	0.328	0.218	0.259	0.271	0.428	0.358	0.421	0.428	0.299	0.356	0.421	/	0.299	0.390	0.000	0.257	0.296	0.259	0.218	0.290	0.390
I-3	0.335	0.351	0.312	0.333	0.337	0.323	0.354	0.340	0.271	0.276	0.287	0.305	0.269	0.413	0.339	0.330	0.413	0.270	0.359	0.330	0.299	/	0.263	0.299	0.262	0.261	0.305	0.287	0.152	0.263
M-1	0.365	0.382	0.359	0.358	0.365	0.354	0.386	0.381	0.336	0.333	0.375	0.417	0.401	0.453	0.393	0.404	0.453	0.328	0.400	0.404	0.390	0.263	/	0.390	0.373	0.370	0.417	0.375	0.295	0.000
M-2	0.399	0.424	0.236	0.383	0.394	0.381	0.421	0.406	0.320	0.328	0.218	0.259	0.271	0.428	0.358	0.421	0.428	0.299	0.356	0.421	0.000	0.299	0.390	/	0.257	0.296	0.259	0.218	0.290	0.390
M-3	0.399	0.415	0.339	0.393	0.396	0.391	0.423	0.412	0.316	0.338	0.209	0.251	0.272	0.434	0.370	0.371	0.434	0.328	0.384	0.371	0.257	0.262	0.373	0.257	/	0.268	0.251	0.209	0.299	0.373
M-4	0.388	0.418	0.358	0.388	0.397	0.370	0.410	0.401	0.320	0.336	0.301	0.313	0.276	0.463	0.395	0.404	0.463	0.361	0.396	0.404	0.296	0.261	0.370	0.296	0.268	/	0.313	0.301	0.295	0.370
M-5	0.426	0.452	0.355	0.403	0.410	0.404	0.431	0.425	0.342	0.362	0.147	0.000	0.238	0.467	0.400	0.433	0.467	0.347	0.400	0.433	0.259	0.305	0.417	0.259	0.251	0.313	/	0.147	0.301	0.417
M-6	0.435	0.445	0.340	0.409	0.423	0.400	0.439	0.420	0.345	0.372	0.000	0.147	0.290	0.445	0.379	0.410	0.445	0.310	0.374	0.410	0.218	0.287	0.375	0.218	0.209	0.301	0.147	/	0.279	0.375
M-7	0.300	0.307	0.262	0.282	0.289	0.275	0.325	0.305	0.216	0.221	0.279	0.301	0.282	0.349	0.272	0.347	0.349	0.232	0.296	0.347	0.290	0.152	0.295	0.290	0.299	0.295	0.301	0.279	/	0.295
M-8	0.365	0.382	0.359	0.358	0.365	0.354	0.386	0.381	0.336	0.333	0.375	0.417	0.401	0.453	0.393	0.404	0.453	0.328	0.400	0.404	0.390	0.263	0.000	0.390	0.373	0.370	0.417	0.375	0.295	/

Supplementary Table S4 Per-sample and per-chromosome missingness in the ALL, HOM, and FINAL SNP datasets.

MISSING_RATE_PER_CHROMOSOME			
CHROMOSOME	ALL_DATASET	FINAL_DATASET	HOM_DATASET
1	0.30	0.32	0.29
2	0.30	0.33	0.29
3	0.30	0.32	0.30
4	0.30	0.32	0.28
5	0.28	0.34	0.29
6	0.30	0.34	0.32
7	0.30	0.33	0.29
8	0.30	0.32	0.27
9	0.30	0.33	0.29
10	0.31	0.33	0.28

MISSING_RATE_PER_SAMPLE			
SAMPLE	ALL_DATASET	FINAL_DATASET	HOM_DATASET
L-1	0.26	0.35	0.25
L-2	0.28	0.42	0.29
L-3	0.51	0.53	0.46
L-4	0.44	0.45	0.42
L-5	0.40	0.43	0.37
L-6	0.32	0.33	0.30
L-7	0.30	0.33	0.29
L-8	0.63	0.67	0.63
L-9	0.64	0.67	0.62
Mo17	0.40	0.42	0.38
B73	0.24	0.26	0.24
B-1	0.21	0.22	0.20
B-2	0.23	0.24	0.21
B-3	0.13	0.16	0.16
B-4	0.34	0.40	0.36
B-5	0.15	0.17	0.17
B-6	0.13	0.16	0.16
B-7	0.49	0.51	0.45
B-8	0.19	0.20	0.18

I-1	0.15	0.17	0.17
I-2	0.22	0.24	0.22
I-3	0.28	0.41	0.28
M-1	0.23	0.24	0.21
M-2	0.22	0.24	0.22
M-3	0.19	0.21	0.17
M-4	0.18	0.18	0.16
M-5	0.21	0.22	0.20
M-6	0.24	0.26	0.24
M-7	0.43	0.48	0.43
M-8	0.23	0.24	0.21
